# There is still room for improvement in the completeness of abstract reporting according to the PRISMA-A checklist: a cross-sectional study on systematic reviews in periodontology

**DOI:** 10.1186/s12874-021-01223-y

**Published:** 2021-02-11

**Authors:** Milagros Adobes Martin, Sala Santamans Faustino, Inmaculada Llario Almiñana, Riccardo Aiuto, Roberto Rotundo, Daniele Garcovich

**Affiliations:** 1 Department of Dentistry, Universidad Europea de Valencia, Paseo de la Alameda 7, 46010 Valencia, Spain; 2 Department of Dentistry, University of Valencia, Valencia, Spain; 3 Department of Dentistry, Universidad Catolica de Valencia, Valencia, Spain; 4 Department of Oral Rehabilitation, Istituto Stomatologico Italiano, University of Milan, Milan, Italy; 5 Periodontology Unit, Eastman Dental Institute, University College of London, London, UK

**Keywords:** PRISMA, Reporting quality, Abstracts, Systematic reviews, Periodontology

## Abstract

**Background:**

To evaluate the completeness of reporting abstracts of systematic reviews (SRs) before and after the publication of the PRISMA-A checklist in 2013 and to assess if an association exists between abstract characteristics and the completeness of reporting.

**Methods:**

A systematic search of the literature was conducted in the PubMed and Scopus databases in March 2020. The search focused on the SRs of evaluations of interventions published since 2002 in the field of periodontology. The abstracts of the selected SRs were divided into two groups before and after publication of the PRISMA-A checklist in 2013, and compliance with the 12 items reported in the checklist was evaluated by three calibrated evaluators.

**Results:**

A set of 265 abstracts was included in the study. The total score before (mean score, 53.78%; 95% CI, 51.56–55.90%) and after (mean score, 56.88%; 95% CI, 55.39–58.44%) the publication of the PRISMA-A statement exhibited a statistically significant improvement (*P* = 0.012*). Nevertheless, only the checklist items *included studies* and *synthesis of the results* displayed a statistically significant change after guideline publication. The total PRISMA-A score was higher in the meta-analysis group and in articles authored by more than four authors.

**Conclusions:**

The impact of the PRISMA-A was statistically significant, but the majority of the items did not improve after its introduction. The editors and referees of periodontal journals should promote adherence to the checklist to improve the quality of the reports and provide readers with better insight into the characteristics of published studies.

**Supplementary Information:**

The online version contains supplementary material available at 10.1186/s12874-021-01223-y.

## Background

Systematic reviews (SRs) and meta-analyses (MAs) are located at the top of the scientific evidence pyramid, offering the highest level of scientific evidence on clinical protocols and diagnostic procedures [1]. They are one of the most cited published items and are used by research stakeholders to appraise, synthetize, and apply evidence [2]. Notably, SRs employ a study design that attracts more online attention and accrue great interest outside academia [3]. Scientific production has experienced a fast increase in recent decades, with a current production of more than 2.5 billion articles, doubling every 9 years [4]. The number of published systematic reviews displayed a similar trend and increased sharply in recent decades in both dentistry and medicine [5, 6]. A behaviour analysis of average PubMed users highlighted how most of them only looked at titles. When an abstract was requested and viewed, the user moved on and retrieved the corresponding full-text article only 29% of the time. Indeed, after the title, the abstract is the most read part of a biomedical article [7]. An abstract can either be the only part of a published item accessed by the reader or be the article section used to determine whether to review the full text. The abstracts of SRs should therefore provide a structured summary that allows a quick appraisal of the validity and applicability of the review and an easy retrieval after an electronic search. In 2013, the Preferred Reporting Items for Systematic Reviews and Meta-Analyses (PRISMA) steering group published consensus-based reporting guidelines as an extension to the PRISMA statement to improve the quality of reporting of SRs in journals and conference abstracts. The PRISMA for Abstracts (PRISMA-A) checklist provides the authors with a framework to summarize the essential sections of their systematic review to meet the needs of the readers [8]. Although the effect of the PRISMA-A has been evaluated in the medical literature, this was not done in periodontal literature, and its effect on the reviews published in this branch of dentistry is still unclear. The aims of this research were to evaluate and compare the quality of reporting of abstracts of SRs before and after the publication of the PRISMA-A in 2013 and to evaluate whether an association exists between the abstract characteristics and the completeness of reporting, as measured by the PRISMA-A checklist.

## Methods

### Eligibility criteria

Articles on a periodontics-related topic with the term “systematic review” or “meta-analysis” in their title, abstract, or keywords were included. The search was limited to items published in the English language from 2002 to 2020. This time window was chosen considering that 2009 was the year of publication of the PRISMA statement [9], whereas in 2013, the PRISMA for abstracts guidelines was published [8].

Systematic reviews of evaluations of interventions were included. Systematic reviews incorporating the aetiology, diagnostics, test accuracy, or prognosis were excluded. All primary studies were also excluded. Cochrane reviews were excluded because they adhered to their own reporting standards [10].

### Search strategy

A systematic search of the literature was conducted independently and in duplicate by 2 authors (DG and RA) using the PubMed and Scopus electronic databases in March 2020.

First, the search was performed in both databases using MeSH terms related to the field of periodontics and provided by the United States National Library of Medicine (https://meshb.nlm.nih.gov/record/ui?ui=D003813). According to the MeSH tree structure, the MeSH terms related to the field of periodontics [E06.721] belong to a specific subcategory of dentistry mesh terms descriptors [E06]. The MeSH terms used were dental prophylaxis [E06.721.189]; dental scaling [E06.721.189.350]; periodontal debridement [E06.721.189.675]; gingivectomy [E06.721.321]; gingivoplasty [E06.721.384]; guided tissue regeneration, periodontal [E06.721.485]; periodontal dressings [E06.721.595]; periodontal index [E06.721.658]; periodontal prosthesis [E06.721.721]; periodontal splints [E06.721.721.680]; subgingival curettage [E06.721.874]; and root planing [E06.721.874.650].

A second search was then performed to find the systematic reviews and meta-analyses published in periodontics journals listed in the 2018 edition of the Journal Citation Report (JCR) in the category “Dentistry Oral Surgery and Medicine”.

The journals included in the search were The Journal of Clinical Periodontology, the Journal of Periodontology, Periodontology 2000, the Journal of Periodontal Research, the Journal of Periodontal & Implant Science, and the Journal of Periodontics and Restorative Dentistry. The journals with a specific focus on periodontology were targeted by a specific search because, according to our previous experience in the bibliometric field, performing such a search can improve overall search efficiency [2, 3, 11].

The search strings used in the advanced search tool of the PubMed and Scopus databases are reported in Table 1.

**Table 1:** The search strings inserted in the advanced search tool of PubMed and Scopus. The date of the search and the retrieved items are presented

Database	SEARCH STRING
**PubMed** *Search 1*	(((((((((((((((dental prophylaxis [MeSH terms]) OR dental scaling [MeSH terms]) OR periodontal debridement [MeSH terms]) OR gingivectomy [MeSH terms]) OR gingivoplasty [MeSH terms]) OR guided tissue regeneration, periodontal [MeSH terms]) OR periodontal dressings [MeSH terms]) OR periodontal index [MeSH terms]) OR periodontal prosthesis [MeSH terms]) OR periodontal splints [MeSH terms]) OR subgingival curettage [MeSH terms]) OR root planing [MeSH terms])) AND ((systematic review) OR meta-analysis))) AND (““2002/01/01″“[date - publication]: ““3000″“[date - publication])” Date 18.03.2020, 11:52:51; 466 Items
**PubMed** *Search 2*	((((systematic review) OR meta-analysis)) AND ((((((““Journal of Clinical Periodontology”“[Journal]) OR ““Journal of Periodontology”“[Journal]) OR ““Periodontology 2000″“[Journal]) OR ““Journal of Periodontal Research”“[Journal]) OR ““Journal of Periodontal & Implant Science”“[Journal]) OR (““Journal of Periodontics and Restorative Dentistry”“[Journal]))) AND (““2002/01/01″“[date - publication]: ““3000″“[date - publication])” Date 18.03.2020; 11:52:26, 493 Items
**Scopus** *Search 1*	**(**TITLE-ABS-KEY **(***“dental prophylaxis”* **OR** *“dental scaling”* **OR** *“periodontal debridement”* **OR** *“gingivectomy”* **OR** *“gingivoplasty”* **OR** *“guided tissue regeneration, periodontal”* **OR** *“periodontal dressings”* **OR** *“periodontal index”* **OR** *“periodontal prosthesis”* **OR** *“periodontal splints”***) AND** TITLE-ABS-KEY **(***“systematic review”* **OR** *“meta-analysis”***)) AND** PUBYEAR **>** *2001* **AND (**LIMIT-TO **(**LANGUAGE**,** *“English”*)**)** Date; 18.03.2020 519 Items
**Scopus** *Search 2*	(SRCTITLE (“Journal of Clinical Periodontology” OR “Journal of Periodontology” OR “Periodontology 2000” OR “Journal of Periodontal Research” OR “Journal of Periodontal & Implant Science” OR “Journal of Periodontics and Restorative Dentistry”) AND TITLE-ABS-KEY (“systematic review” OR “meta-analysis”)) AND PUBYEAR > 2001 AND (LIMIT-TO (LANGUAGE, “English”)) Date; 18.03.2020 527 Items

Three researchers (DG, RA, and FS) screened, as a team during two sessions, the retrieved SRs and MAs according to the inclusion and exclusion criteria. The same team of reviewers extracted the information regarding the retrieved items, and in case of disagreement, any conflict was resolved through discussion. The following data were saved on an Excel datasheet (Microsoft Office for Mac 2011 package format): (1) review title; (2) authors; (3) number of authors; (4) type of review (SR or MA); (5) journal title; (6) DOI; (7) year of publication; (8) affiliation of the corresponding author, i.e., university or other; (9) origin of the article (as defined by the corresponding author), i.e., USA, Canada, Italy, China; (10) number of citations in Scopus; (11) article subject (surgical or nonsurgical treatment); and (11) the presence of a structured or nonstructured abstract. Data are available in Additional file 1. The abstracts of the selected SRs and MAs were stored and made available through an online Mendeley folder (Mendeley desktop 1.19.4 for MacOS 2020).

To evaluate the impact of the PRISMA-A release, the included studies were divided into two groups according to the year of publication (2002–2013 vs 2014–2020).

The impact of the review type (SR or MA), the number of authors, the country of origin, the article subject, and the abstract structure on the completeness of the report were explored.

### Assessment of reporting completeness

The abstracts of the selected articles were evaluated using the 12 items of the PRISMA-A checklist [8] (Table 2). Each item was evaluated on a scale from 0 to 2, where 0 meant that the item was not reported at all, 1 meant that the item was only partially or inadequately reported, and 2 meant the item was fully reported. A higher score, therefore, denoted higher quality reporting, and the highest possible score was 24 points. The reference guide used for abstract scoring is provided in Additional file 2.

**Table 2:** The Prisma for abstracts checklist as presented by Beller et al. in 2013

Section/topic	#	PRISMA for Abstracts Checklist item
**TITLE and PURPOSE**		
Title	1	Identify the report as a systematic review (+/− meta-analysis) or both.
Objectives	2	Indicate the research question, intervention, comparator and outcomes.
**METHODS**		
Eligibility criteria	3	Include study characteristics used as criteria for eligibility.
Information sources	4	List the key databases searched and the search dates.
Risk of bias & applicability	5	Indicate the methods of assessing risk of bias and applicability.
**RESULTS**		
Included studies	6	Number and type of included studies and the participants and relevant characteristics of studies.
Synthesis of results	7	Results for main outcomes (benefits and harms), preferablyindicating the number of studies and participants for each.If meta-analysis was done, include summary measures and confidence intervals.
Description of the effect	8	Direction of the effect (i.e., which group is favoured) and size ofthe effect in terms meaningful to clinicians and patients.
**DISCUSSION**		
Strengths and limitations	9	Provide a brief summary of the strengths and limitations of the evidence.
Interpretation	10	Provide a general interpretation of the results and the important implications.
**OTHER**		
Funding	11	Indicate the primary source of funding for the review.
Registration	12	Provide the registration number and the registry name.

According to the PRISMA-A guidelines, both SRs and MAs were scored according to the same items, but in the case of MAs in item 7 (*synthesis of results*), summary measures and confidence intervals were be reported, and in item 9 (*strengths and limitations of evidence*), heterogeneity were to be discussed. Failing to report these data in an MA abstract had a negative impact on the completeness of the report [8].

### Training and calibration

Abstracts were assessed independently by three reviewers who had no prior experience with the PRISMA-A (DG, RA, and FS) and who underwent a calibration process prior to the start of the screening. First, they were involved in theoretical training sessions in which the abstracts of 10 SRs were reviewed according to the checklist with the help of supporting documents [8] and the support of other members of the research group (RR) who had previous experience with the PRISMA-A and authored several SRs. The abstracts used for training were not included in the selected pool, and any disagreements were resolved through consensus. Calibration sessions were repeated with sets of 10 abstracts assessed twice with a week between each assessment until excellent intra- and inter-operator reliability was achieved. An intraclass correlation coefficient (ICC) higher than 0.90 was considered enough to define the method error as low and obtain high intra- and inter-operator reliability.

### Statistical analysis

Descriptive models were used to describe ordinal and continuous variables (mean, standard deviation, range, and median). Categorical variables were described by means of absolute and relative frequencies. Due to the large sample size, a 95% confidence interval was obtained for score means. To determine the distribution of the data, the Kolmogorov-Smirnov test was applied for quantitative variables. Nonnormally distributed data were found, and nonparametric tests, which included the Mann-Whitney U test and Kruskal-Wallis test, were used. The reference level of significance was designated as up to 5% (α = 0.05). Post hoc power was calculated, and the final abstract pool provided 97.1% of the statistical power in detecting an effect size d = 0.5 (medium) between groups using Mann-Whitney’s test and assuming a confidence level of 95%.

## Results

In total, 2005 published items were initially retrieved. After the removal of duplicates, 925 articles were retained for further screening. After applying the inclusion and exclusion criteria, a final set of 265 abstracts was included in the study. The included articles were published by 19 journals from 2002 to 2013 and by 43 journals from 2014 to 2020. Only eight journals published and included SRs in both periods, as reported in Tables 3 and 4.

**Table 3:** The 19 journals in which the selected systematic reviews were published from 2002 to 2013. Journals that published and included SRs in both studied periods are presented in boldface

Title	***Reviews published from 2002 to 2013***			
	ISSN	N	Total P-A score	P-A /Item
**Journal of Clinical Periodontology**	0303–6979	40	523.00	13.08
**Journal of Periodontology**	0022–3492	28	366.00	13.07
**International Journal of Dental Hygiene**	1601–5029	7	77.00	11.00
Annals of Periodontology	1553–0841	4	62.00	15.50
Clinical Oral Implants Research	0905–7161	2	27.00	13.50
**Journal of Dentistry**	0300–5712	2	30.00	15.00
Journal of Dental Research	0022–0345	2	26.00	13.00
**Journal of Periodontal Research**	0022–3484	2	27.00	13.50
**Journal of the American Dental Association**	0002–8177	2	29.00	14.50
**Lasers in Medical Science**	0268–8921	2	18.00	9.00
The International Journal of Oral & Maxillofacial Implants	0882–2786	1	10.00	10.00
Acta Odontologica Latinoamericana: AOL	0326–4815	1	8.00	8.00
Journal (Canadian Dental Association)	0709–8936	1	15.00	15.00
International Journal of Oral and Maxillofacial Surgery	0901–5027	1	12.00	12.00
British Dental Journal	0007–0610	1	11.00	11.00
**Evidence-based Dentistry**	1462–0049	1	14.00	14.00
Journal of Oral Science	1343–4934	1	11.00	11.00
Clinical Implant Dentistry and Related Research	1523–0899	1	11.00	11.00
Pediatric Dentistry	0164–1263	1	11.00	11.00

*ISSN* International Standard Serial Number, *N* the number of published SRs or MAs, *P-A* PRISMA-A

**Table 4:** The 43 journals in which the selected articles were published from 2014 to February 2020. Journals that published and included SRs in both studied periods are presented in boldface

Title	***Reviews published from 2014 to 2020***			
	ISSN	N	Total score	P-A /Item
**Journal of Clinical Periodontology**	0303–6979	33	436	13.21
**Journal of Periodontology**	0022–3492	18	250	13.89
Photodiagnosis and Photodynamic Therapy	1572–1000	13	174	13.38
**Journal of Periodontal Research**	0022–3484	12	169	14.08
**International Journal of Dental Hygiene**	1601–5029	11	151	13.73
Clinical Oral Investigations	1432–6981	10	159	15.90
BMC Oral Health	1472–6831	7	106	15.14
**Journal of the American Dental Association**	0002–8177	4	60	15.00
Journal of Investigative and Clinical Dentistry	2041–1618	4	53	13.25
**Evidence-based Dentistry**	1462–0049	4	54	13.50
Photomedicine and Laser Surgery	1549–5418	3	40	13.33
**Journal of Dentistry**	0300–5712	3	44	14.67
Clinical Oral Implants Research	0905–7161	2	33	16.50
**Lasers in Medical Science**	0268–8921	2	22	11.00
Medicine (Baltimore)	0025–7974	2	27	13.50
American Journal of Orthodontics and Dentofacial Orthopedics	0889–5406	2	30	15.00
Journal of Prosthodontics	1059-941X	2	21	10.50
Implant Dentistry	1056–6163	2	23	11.50
Brazilian Oral Research	1806–8324	2	33	16.50
Journal of Applied Oral Science: Revista FOB	1678–7757	2	32	16.00
Journal of Clinical and Diagnostic Research	2249-782X	2	23	11.50
Biomed Research International	2314–6141	2	18	9.00
Quintessence International	0033–6572	2	21	10.50
The International Journal of Periodontics & Restorative Dentistry	0198–7569	2	25	12.50
Diabetes Care	0149–5992	1	15	15.00
European Journal of Orthodontics	0141–5387	1	12	12.00
PLoS ONE	1932–6203	1	14	14.00
Expert Review of Anti-Infective Therapy	1478–7210	1	12	12.00
British Journal of Clinical Pharmacology	0306–5251	1	13	13.00
Biomolecules	2218-273X	1	11	11.00
International Dental Journal	0020–6539	1	14	14.00
Complementary Therapies in Medicine	0965–2299	1	18	18.00
Archives of Oral Biology	0003–9969	1	8	8.00
Journal of Clinical and Experimental Dentistry	1989–5488	1	16	16.00
The Journal of Evidence-based Dental Practice	1532–3382	1	13	13.00
Australian Dental Journal	0045–0421	1	13	13.00
Medical Science Monitor: International Medical Journal of Experimental and Clinical Research	1234–1010	1	17	17.00
Journal of Indian Society of Periodontology	0972-124X	1	14	14.00
Oxidative Medicine and Cellular Longevity	1942–0900	1	15	15.00
Angle Orthodontist	0003–3219	1	10	10.00
Molecules (Basel, Switzerland)	1420–3049	1	10	10.00
Medicina Oral Patologia Oral y Cirugia Bucal	1698–4447	1	13	13.00
American Journal of Dentistry	0894–8275	1	15	15.00

*ISSN* International Standard Serial Number, *N* the number of published SRs or MAs, *P-A* PRISMA-A

**Fig. 1:**
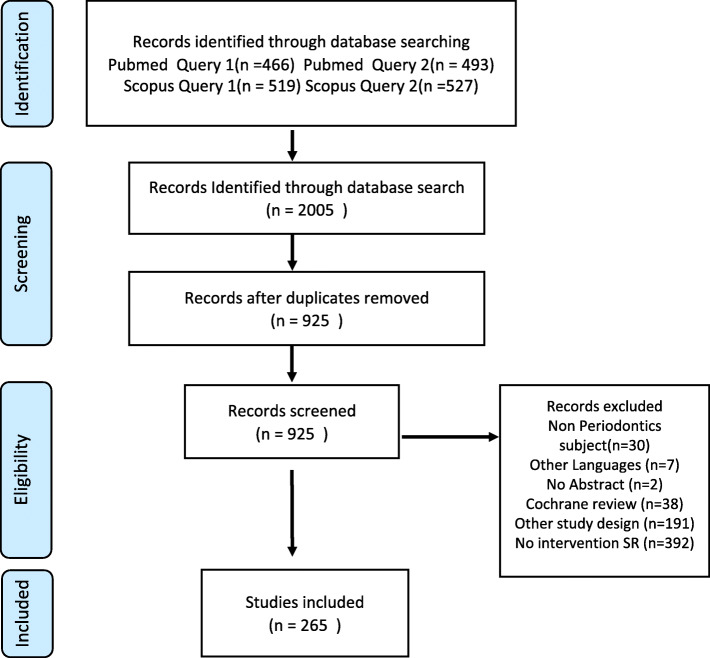
Flow diagram of the studies considered for inclusion

Further details of the search and screening process are shown in the flow diagram in Fig. 1. The list of references of the included studies is available in Additional file 3. The results of the quality assessment of the included SRs are available in Additional file 4.

As outlined in Table 5, the mean general PRISMA-A score was 55.72% (95% CI, 54.46–56.79%). The total score before (mean score, 53.78%; 95% CI, 51.56–55.90%) and after (mean score, 56.88%; 95% CI, 55.39–58.44%) the publication of the PRISMA-A statement revealed a statistically significant improvement (*P* = 0.012*). The most recent pool of abstracts presented a better score. The improvement in the overall score after 2013 was mainly due to the improvement in reporting item number 6 “*included studies*” (*p* = 0.004) and item number 7 “*synthesis of the results*” (*p* = 0.025) of the PRISMA-A checklist. The lowest scores in both groups were those in the “*funding and conflict of interest report*” and “*registration*” sections, followed by “*risk of bias*” and “*strength and limitation of evidence*” sections.

**Table 5:** Comparison of the percentage of studies complying with PRISMA-A items before and after the publication of the checklist. Mann-Whitney U test *p < 0.05; **p < 0.01

	Total (***N*** = 265)		2002–2013 (***N*** = 101)		2014–2020 (***N*** = 165)		
Item	Mean	95% CI	Mean	95% CI	Mean	95% CI	***p***-value
Title	93.23	90.33–96.13	91.58	85.79–96.68	94.24	90.63–97.63	0.346
Objectives	84.96	81.73–88.20	85.64	80.33–90.80	84.55	80.30–88.82	0,827
Eligibility criteria	63.53	58.50–68.57	65.84	56.81–74.11	62.12	55.69–68.37	0.368
Information sources	62.22	57.22–67.20	58.42	49.80–66.68	64.55	58.17–70.84	0.23
Risk of bias	16.17	12.03–20.29	13.86	8.09–20.76	17.58	12.25–23.54	0.596
Included studies	69.43	65.58–73.28	62.00	55.70–69.04	73.94	69.09–78.43	0.004**
Synthesis of results	76.88	72.88–80.87	70.79	62.73–77.47	80.61	76.11–85.61	0.025*
Description of the effect	77.07	72.93–81.19	76.73	69.93–82.64	77.27	71.55–82.77	0.472
Strengths and limitations of the evidence	32.52	27.48–37.55	30.20	21.84–37.95	33.94	27.01–40.27	0.561
Interpretation	93.05	90.58–95.50	92.08	87.22–96.27	93.64	90.86–96.79	0.614
Funding and conflict of interest	0	–	0	–	0	–	1
Registration	0.38	0.00–1.12	0	–	0.61	0.00–1.83	0.434
Total PRISMA-A score	55.72	54.46–56.97	53.78	51.56–55.99	56.88	55.39–58.44	0.012*

*CI* confidence interval, *N* number of published SRs and MAs

**Fig. 2:**
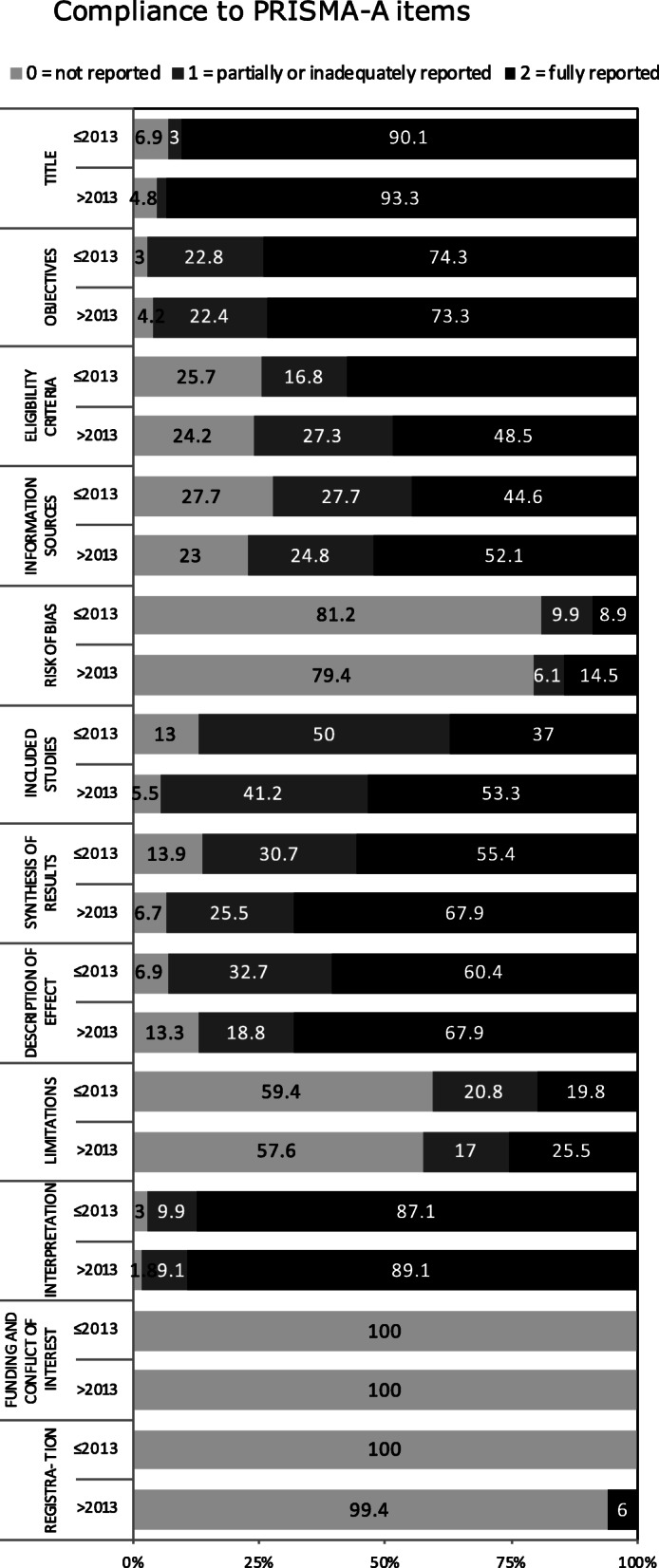
The distribution of compliance with items on the PRISMA for abstracts checklist according to the publication periods. Score: 0 = not reported, 1 = partially or inadequately reported, and 2 = fully reported

The distribution of compliance with items in the PRISMA-A checklist according to the publication period is graphically presented in Fig. 2.

As highlighted in Table 6, only the International Journal of Dental Hygiene displayed a significant improvement in the PRISMA-A score when comparing the two studied periods, whereas top-cited journals such as the Journal of Clinical Periodontology or the Journal of Periodontology did not show any improvement.

**Table 6:** Comparison of PRISMA-A items by journal before and after the publication of the checklist. Mann-Whitney U test *p < 0.05; **p < 0.01

	***2002–2013***			***2014–2020***			
	N	Total score	P-A /Item	N	Total score	P-A /Item	p
Journal of Clinical Periodontology	40	523	13.08	33	436	13.21	0.826
Journal of Periodontology	28	366	13.07	18	250	13.89	0.177
Journal of Periodontal Research	2	27	13.5	12	169	14.08	0.39
International Journal of Dental Hygiene	7	77	11	11	151	13.73	0.022*
Journal of the American Dental Association	2	29	14.5	4	60	15	0.5
Journal of Dentistry	2	30	15	3	44	14.67	0.6
Lasers in Medical Science	2	18	9	2	22	11	0.333
Evidence-based Dentistry	1	3	14	4	54	13.5	N/A

*N* the number of published SRs or MAs, *P-A* PRISMA-A

Half of the selected articles were systematic reviews without meta-analysis (51.3%). This percentage dropped over time (64% before 2014 and 43.6% from 2014 to 2020). As reported in Table 7, the total PRISMA-A score was significantly higher in the meta-analysis group. The mean number of authors increased over time, from 3.9 authors prior to 2014 to 4.9 authors in SRs/MAs published after 2013. The total PRISMA-A score was significantly higher in the articles authored by more than four authors.

**Table 7:** Comparison of the percentage of studies complying with PRISMA-A items according to the type of review and the number of authors. Mann-Whitney U test *p < 0.05; **p < 0.01; ***p < 0.001

	TYPE OF REVIEW					NUMBER OF AUTHORS				
	MAs (***n*** = 129)		SRs (***n*** = 136)			1–4 (***n*** = 140)		> 4 (***n*** = 125)		
**ITEM**	**Mean**	**95% CI**	**Mean**	**95% CI**	**p**	**Mean**	**95% CI**	**Mean**	**95% CI**	**p**
Title	91.86	87.14–96.58	94.53	91.02–98.02	0.655	90.43	85.76–95.08	96.4	93.18–99.61	0.028*
Objectives	84.5	79.77–89.21	85.4	80.91–89.88	0.771	82.62	77.85–87.39	87.6	83.29–91.90	0.153
Eligibility criteria	64.73	57.55–71.90	62.41	55.25–69.56	0.68	60.28	53.35–67.21	67.2	59.81–74.58	0.14
Information sources	58.53	51.29–65.75	65.69	58.76–72.62	0.139	64.18	57.41–70.95	60	52.54–67.45	0.428
Risk of bias	21.71	14.83–28.57	10.95	6.28–15.61	0.030*	12.41	7.22–17.59	20.4	13.83–26.96	0.035*
Included studies	73.26	67.69–78.81	65.81	60.47–71.14	0.038*	63.21	57.85–68.57	76.4	71.05–81.74	0.001**
Synthesis of results	84.5	79.65–89.34	69.71	63.61–75.79	< 0.001***	73.76	67.99–79.52	80.4	74.90–85.89	0.106
Description of the effect	85.27	80.23–90.30	69.34	63.09–75.59	< 0.001***	75.53	69.76–81.30	78.8	72.82–84.77	0.375
Strengths and limitations of the evidence	27.91	20.77–35.03	36.86	29.74–43.97	0.054	31.91	25.00–38.82	33.2	25.74–40.65	0.81
Interpretation	93.02	89.13–96.91	93.07	89.96–96.17	0.506	92.55	89.11–95.99	93.6	90.04–97.15	0.564
Funding and conflict of interest	0	–	0	–	1	0	–	0	–	1
Registration	0.78	0.00–2.30	0	–	0.303	0	–	0.8	0.00–2.38	0.288
**Total PRISMA-A score**	57.11	55.26–58.95	54.41	52.69–56.12	0.026*	53.77	52.06–55.48	57.89	56.08–59.69	< 0.001***

*CI* confidence interval, *N* number of published SRs or MAs, *SRs* systematic review, *MA* meta-analyses

European institutions were the most prevalent affiliation of the first author (47.2%), followed by North America (15.5%), Asia (15.1%), and Latin America (14.3%). Latin America and North America turned out to be the most compliant areas in terms of abstract reports, and these are the areas that increased their relative prevalence more after 2013 (Additional file 5). A structured abstract was present in 87.2% of the cases and was related to a higher PRISMA-A score when compared to nonstructured abstract items. The difference was not statistically significant.

## Discussion

To the best of our knowledge, this is the first study to address the completeness of abstract reporting in SRs in periodontology using the PRISMA-A checklist, since previously published studies about abstract completeness in periodontology and oral implantology were performed prior to the publication of the checklist in 2013 with different assessment methods of the included studies [12, 13].

The present study provided analyses of a larger data pool compared to previous investigations in the same branch of dentistry. A similar article was recently published in Orthodontics, including 389 abstracts [14] of SRs on intervention and non-intervention procedures. The PRISMA-A checklist was designed with a special focus on systematic reviews of evaluations of interventions in which one or more meta-analyses were conducted [8]. If SRs are performed on non-intervention protocols or on questions about aetiology or diagnostic test accuracy, there may be a need to modify items or include others to improve the quality of the report. In the present study, non-intervention SRs were excluded to reduce possible bias in the assessment process.

The primary objective of this study was to assess the impact of the PRISMA-A on the completeness of abstract reporting in SRs. The results highlighted how the total score presented a statistically significant improvement after guideline publication.

However, a discussion of the results based only on the total score could be misleading since it could omit important information. Despite the global improvement, the analysis of the scores obtained by each of the checklist items highlights how 10 out of 12 items did not exhibit a statistically significant change after guideline publication. The leading periodontal journals, such as the Journal of Clinical Periodontology or the Journal of Periodontology, did not show any improvement in the completeness of the report, and although they require authors of SRs to comply with the PRISMA guidelines, the journals do not seem to have implemented the PRISMA-A checklist and guidelines effectively.

Other authors in different fields of dentistry also considered compliance with the guidelines to be poor [14, 15]. Faggion et al. in 2013 suggested how the authors of SRs with meta-analyses should improve the report of the limitations of evidence, the risk of bias, and the measures of heterogeneity among the included primary studies to enable the readers to understand the strengths and weaknesses of the findings and allow for better clinical application of the evidence. These findings were consistent with those of our study that outlined how “risk of bias” and “strength and limitation of evidence” were some of the less reported items. In 2019, in an appraisal of the SRs published in top-ranked nursing journals, Wang et al. found no difference in completeness before and after the PRISMA-A guideline release [16]. Maticic et al., in the field of anaesthesiology, and Bigna et al., in the field of general medicine, reported poor or no improvement after checklist publication [17]. In agreement with our results, other authors in different fields of medicine and dentistry reported how information about registration is often missing in SRs´ abstracts [13–16, 18]. The lack of registration reports is surprising, especially in the SRs published after 2013, taking into account the wide acceptance of registration databases such as PROSPERO, which from its launch in 2011 to 2017 registered more than 30,000 SRs [19]. It is important to note that even if the SRs were not registered, this should have been reported in the abstract. A similar consideration may also be applied to information about funding, which in our pool was not reported in any abstract.

According to our findings, a higher number of authors is related to a better quality report, and the same result was also highlighted by previous research [13, 14, 16, 18]. In contrast, Bigna et al. (2016), in top-rated medical journals, reported no association between the number of authors and the quality of the report [20]. However, the authors discriminated between articles with more or fewer than nine authors since the number of co-authors is higher in medicine than in dentistry. The correlation between the quality of the report and the number of authors could be due to the nature of SRs. Writing a systematic review involves a lengthy and elaborate process following strict adherence to search, screening, and selection protocols. From this perspective, relying on a larger study group can ensure better support and collaboration, favouring higher quality research output. According to the data presented by previous reports, the MAs group displayed better compliance with the PRISMA-A checklist than the SRs group [13, 14, 18]. It should be noted that the number of MAs was higher among the most recently published items that generally presented higher scores. According to what was reported by other authors in bibliometric studies about SRs [13, 16, 18, 21], the majority of first authors belonged to European institutions. However, other articles, such as that by Wasiak et al. [22], had a higher prevalence of North American first authors, whereas the most prevalent authors were from Latin America in Bassani et al. [23]. It should be noted that the report by Bassani et al. [23] only included SRs published during 2017, and their findings cannot be generalized to all SRs throughout time.

Among the 265 abstracts included in the assessment, 34 were not structured, and the prevalence was higher than that previously reported by other authors [13, 18]. Structured abstracts have been strongly recommended by the International Committee of Medical Journal Editors [24], and they are supposed to enhance the quality of the report [25].

The accuracy of the search strategy, the high number of included abstracts, and the accurate training and calibration process of the research group to maximize the accuracy and reliability of the assessment can be considered strengths of this study. A number of limitations of this study should also be acknowledged. The impact of the abstract word count was not explored. In theory, a larger abstract could give more room for a detailed report of the required items. Nevertheless, the relationship of this factor with the quality of the report remains controversial [26]. The full texts of the selected articles were not systematically assessed and thus were not used to detect which items were not reported due to their absence (e.g., funding, conflict of interest or registration) or to a lack of compliance in the report. The lack of a full-text assessment could have also hindered the articles in which the word meta-analysis was not mentioned in the title, abstract, or keywords, in cases in which a meta-analysis was performed as a statistical method. Moreover, in the search strategy, a specific screening was performed among the periodontal journals indexed in the 2018 edition of the JCR, not including the journals indexed in larger databases such as Scopus.

## Conclusions

The statistically significant improvement in the overall PRISMA-A score was largely driven by improvements in only 2 of the 12 checklist items. The reporting of the majority of items did not improve after the introduction of the PRISMA-A checklist in 2013. There is definitely room for further improvement, and efforts should be made to reach the required standards. In particular, “risk of bias”, “strength and limitation of evidence”, “registration” and “funding” are the items that displayed the major need for improvement.

The leading periodontal journals did not show any improvement in the completeness of the report during the studied period.

The editors and referees of periodontal journals should promote adherence to the PRISMA-A checklist to increase the quality of the reports and provide readers with better insight into the study outcomes.

## Supplementary Information

**Additional file 1.** Bibliometric data extracted from the selected SRs.

**Additional file 2.** The reference guide used for abstract scoring.

**Additional file 3.** References of the 265 SRs whose abstracts were included in the study.

**Additional file 4.** The results of the quality assessment of the 265 SR abstracts included in the study.

**Additional file 5.** PRISMA-A item compliance by geographical area.

## Data Availability

All data generated or analysed during this study are included in this published article and its supplementary information files.

## Abbreviations

SRs: Systematic reviews
PRISMA: the Preferred Reporting Items for Systematic Reviews and Meta-Analyses
PRISMA-A: PRISMA for abstracts;
PICO: Patient/Population/Problem, Intervention, Comparison, and Outcome
CI: Confidence interval
ICC: Intraclass correlation coefficient
ISSN: International Standard Serial Number
MAs: Meta-analyses
MeSH: Medical subject headings
