# Supplementary material for: There is still room for improvement in the completeness of abstract reporting according to the PRISMA-A checklist: a cross-sectional study on systematic reviews in periodontology
Source: BMC Med Res Methodol. 2021 Feb 11;21:33. doi: 10.1186/s12874-021-01223-y (PMC7879697; doi:10.1186/s12874-021-01223-y)
Supplement: Supplementary file 2 — Additional file 2. The reference guide used for abstract scoring. [file 12874_2021_1223_MOESM2_ESM.docx]

| **ITEM** | | **SCORE** | **CRITERIA** |
| --- | --- | --- | --- |
| **1** | **Title** | **2** | The title clearly Identifies the report as a systematic review, meta-analysis, or both. |
|  |  | **1** | The title Identifies the report as a systematic review, but fails to report that a meta-analysis was conducted. |
|  |  | **0** | The title does not Identify the report as a systematic review, meta-analysis. |
|  |  |  |  |
| **2** | **Objectives** | **2** | The objectives of the review are clearly stated. The participants, interventions, comparators, outcomes, and study designs are described |
|  |  | **1** | The abstract describes only some of the following items but not all of them: participants; interventions; comparators; outcomes; study designs. |
|  |  | **0** | Neither of the following items are described in the abstract text: participants, interventions, comparators, outcomes, and study designs. |
|  |  |  |  |
| **3** | **Eligibility criteria** | **2** | The inclusion and exclusion criteria are clearly stated in the abstract text. |
|  |  | **1** | Only the inclusion or exclusion criteria are reported or both are reported but partially. Year of publication, publication status and language are not compulsory items to include and should not be taken into account while scoring. |
|  |  | **0** | No inclusion or exclusion criteria are reported in the abstract text. |
|  |  |  |  |
| **4** | **Information sources** | **2** | The abstract describes the key databases searched, and the date range or date of last search. If there are three or fewer databases, all them are listed; otherwise at least three of them should be listed. |
|  |  | **1** | The abstract misses to describe the key databases searched or the date range or date of last search. According to the database information, If the study involved three or fewer databases, and not all are listed or if included more than three but less then three of them are listed. |
|  |  | **0** | The abstract does not describe the key databases searched, and the date range or date of last search. |
|  |  |  |  |
| **5** | **Risk of bias** | **2** | The abstract describes the methods used to assess the risk of bias in the included studies, a description of the individual methodological components assessed is preferred but scales, indexes or checklist are acceptable. |
|  |  | **1** | The abstract describes only some of methods used to assess the risk of bias in the included studies if more than one are used. |
|  |  | **0** | The abstract does not describe any methods used to assess the risk of bias in the included studies. |
|  |  |  |  |
| **6** | **Included studies** | **2** | The number, number of participants and characteristics of the included studies are described in the abstract. |
|  |  | **1** | The abstract does not describe one or two of the following items: number of studies; number of participants; characteristics of the included studies. |
|  |  | **0** | The number of studies, number of participants and characteristics of the included studies are not described in the abstract. |
|  |  |  |  |
| **7** | **Synthesis of results.** | **2** | The abstract describes the results for main outcomes (benefits and harms). Indicating the number of studies and participants for each aspect is preferred but not compulsory and should not be taken into account while scoring . If a meta-analysis was performed the summary measures and confidence intervals are indicated. |
|  |  | **1** | The abstract describes partially the results for main outcomes (only the benefits and not the harms or vice versa). If meta-analysis was performed the summary measures and confidence intervals are not fully reported. |
|  |  | **0** | The abstract does not describe the results for main outcomes (benefits and harms). If meta-analysis was performed the summary measures and confidence intervals are not indicated. |
|  |  |  |  |
| **8** | **Description of effect** | **2** | The abstract indicates the direction of the effect (e.g., lower, fewer, reduced; greater, more, increased) and which group is favoured and the size of the effect using familiar units in terms meaningful to patients and clinicians. |
|  |  | **1** | The abstract partially indicates the direction of the effect (e.g., lower, fewer, reduced; greater, more, increased) or miss to describe which group is favoured or the size of the effect using familiar units in terms meaningful to patients and clinicians. |
|  |  | **0** | The abstract does not describe the direction of the effect. |
|  |  |  |  |
| **9** | **Strengths and limitations of evidence** | **2** | The abstract includes a brief summary of strength and limitations of evidence (e.g., inconsistency, imprecision, indirectness, or risk of bias, other supporting or conflicting evidence). |
|  |  | **1** | The abstract includes a brief summary of strength or limitations of evidence but fails to report all of them. |
|  |  | **0** | The abstract does not include a brief summary of strength and limitations of evidence (e.g., inconsistency, imprecision, indirectness, or risk of bias, other supporting or conflicting evidence). |
|  |  |  |  |
| **10** | **General interpretation of the results and important implications** | **2** | The abstract provides an overall summary of the main effects (positive or negative). If there is insufficient evidence to answer the review’s question, this should be stated. When the results are not statistically significant, authors should distinguish between those where there is insufficient evidence to rule out a difference between treatments (wide confidence interval), and those which have sufficient evidence that an important difference is unlikely (narrow confidence interval). Possible implications for policy and practice should be stated. |
|  |  | **1** | The abstract provides an incomplete summary of the main effects (positive or negative). If there is insufficient evidence to answer the review’s question, does not state it. When the results are not statistically significant, authors does not distinguish between those where there is insufficient evidence to rule out a difference between treatments (wide confidence interval), and those which have sufficient evidence that an important difference is unlikely (narrow confidence interval). Possible implications for policy and practice are not or only partially stated. |
|  |  | **0** | The abstract does not provide an overall summary of the main effects (positive or negative). |
|  |  |  |  |
| **11** | **Funding** | **2** | The abstract includes the main source of funding for the systematic review, whether from host institutions or from external bodies. The abstract includes a statement declaring that no funds were received. |
|  |  | **0** | The abstract does not include information about funding. |
|  |  |  |  |
| **12** | **Registration** | **2** | The abstract includes the number of registration in the pertinent database. The abstract includes a statement declaring that the review was not registered. |
|  |  | **0** | The abstract does not include information about registration. |
