# Supplementary material for: There is still room for improvement in the completeness of abstract reporting according to the PRISMA-A checklist: a cross-sectional study on systematic reviews in periodontology
Source: BMC Med Res Methodol. 2021 Feb 11;21:33. doi: 10.1186/s12874-021-01223-y (PMC7879697; doi:10.1186/s12874-021-01223-y)
Supplement: Supplementary file 3 — Additional file 3. References of the 265 SRs whose abstracts were included in the study. [file 12874_2021_1223_MOESM3_ESM.docx]

| 1. Abduljabbar T, Javed F, Shah A, Samer MS, Vohra F, Akram Z. Role of lasers as an adjunct to scaling and root planing in patients with type 2 diabetes mellitus: a systematic review. Lasers Med Sci. 2017;32:449–59. |
| --- |
| 2. Abduljabbar T, Vohra F, Javed F, Akram Z. Antimicrobial photodynamic therapy adjuvant to non-surgical periodontal therapy in patients with diabetes mellitus: A meta-analysis. Photodiagnosis Photodyn Ther. 2017;17:138–46. |
| 3. Addy M, Moran J, Newcombe RG. Meta-analyses of studies of 0.2% delmopinol mouth rinse as an adjunct to gingival health and plaque control measures. J Clin Periodontol. Division of Restorative Dentistry (Perio), University of Bristol Dental School, Lower Maudlin Street, Bristol BS1 2LY, United Kingdom; 2007;34:58–65. |
| 4. Aghaloo TL, Moy PK. Which hard tissue augmentation techniques are the most successful in furnishing bony support for implant placement? Int J Oral Maxillofac Implants. United States; 2007;22 Suppl:49–70. |
| 5. Akram Z, Vohra F, Javed F. Locally delivered metformin as adjunct to scaling and root planing in the treatment of periodontal defects: A systematic review and meta-analysis. J Periodontal Res. United States; 2018;53:941–9. |
| 6. Akram Z, Abduljabbar T, Sauro S, Daood U. Effect of photodynamic therapy and laser alone as adjunct to scaling and root planing on gingival crevicular fluid inflammatory proteins in periodontal disease: A systematic review. Photodiagnosis Photodyn Ther. 2016;16:142–53. |
| 7. Akram Z, Abduljabbar T, Kellesarian SV, Abu Hassan MI, Javed F, Vohra F. Efficacy of bisphosphonate as an adjunct to nonsurgical periodontal therapy in the management of periodontal disease: a systematic review. Br J Clin Pharmacol. 2017;83:444–54. |
| 8. Akram Z, Al-Shareef SAA, Daood U, Asiri FY, Shah AH, AlQahtani MA, et al. Bactericidal Efficacy of Photodynamic Therapy Against Periodontal Pathogens in Periodontal Disease: A Systematic Review. Photomed Laser Surg. United States; 2016;34:137–49. |
| 9. Akram Z, Hyder T, Al-Hamoudi N, Binshabaib MS, Alharthi SS, Hanif A. Efficacy of photodynamic therapy versus antibiotics as an adjunct to scaling and root planing in the treatment of periodontitis: A systematic review and meta-analysis. Photodiagnosis Photodyn. Ther. 2017. p. 86–92. |
| 10. Akram Z, Raffat MA, Saad Shafqat S, Mirza S, Ikram S. Clinical efficacy of photodynamic therapy as an adjunct to scaling and root planing in the treatment of chronic periodontitis among cigarette smokers: A systematic review and meta-analysis. Photodiagnosis Photodyn Ther. Netherlands; 2019;26:334–41. |
| 11. Akram Z, Vohra F, Javed F. Efficacy of statin delivery as an adjunct to scaling and root planing in the treatment of chronic periodontitis: A meta-analysis. J Investig Clin Dent. Department of Periodontology, Faculty of Dentistry, Ziauddin University, Karachi, Pakistan; 2018;9:e12304. |
| 12. Al Amri MD, Kellesarian SV. Crestal Bone Loss Around Adjacent Dental Implants Restored with Splinted and Nonsplinted Fixed Restorations: A Systematic Literature Review. J Prosthodont. United States; 2017;26:495–501. |
| 13. Al-Hamdan K, Eber R, Sarment D, Kowalski C, Wang H-L. Guided tissue regeneration-based root coverage: meta-analysis. J Periodontol. United States; 2003;74:1520–33. |
| 14. Al-Hamoudi N. Is antimicrobial photodynamic therapy an effective treatment for chronic periodontitis in diabetes mellitus and cigarette smokers: a systematic review and meta-analysis. Photodiagnosis Photodyn Ther. Netherlands; 2017;19:375–82. |
| 15. Albaker AM, ArRejaie AS, Alrabiah M, Abduljabbar T. Effect of photodynamic and laser therapy in the treatment of peri-implant mucositis: A systematic review. Photodiagnosis Photodyn Ther. Netherlands; 2018;21:147–52. |

Additional file 3. continued

| 16. Alberton Nuernberg MA, Janjacomo Miessi DM, Ivanaga CA, Bocalon Olivo M, Ervolino E, Gouveia Garcia V, et al. Influence of antimicrobial photodynamic therapy as an adjunctive to scaling and root planing on alveolar bone loss: A systematic review and meta-analysis of animal studies. Photodiagnosis Photodyn Ther. Netherlands; 2019;25:354–63. |
| --- |
| 17. Aljudaibi S, Duane B. Do adjunctive statins improve periodontal treatment outcomes in patients with chronic periodontitis? Evid. Based. Dent. 2019. p. 18–9. |
| 18. AlKudmani H, Al Jasser R, Andreana S. Is Bone Graft or Guided Bone Regeneration Needed When Placing Immediate Dental Implants? A Systematic Review. Implant Dent. United States; 2017;26:936–44. |
| 19. Ambrosio LMB, Rovai ES, Sendyk DI, Holzhausen M, Pannuti CM, Ambrósio LMB, et al. Does the adjunctive use of statins provide additional benefits to nonsurgical periodontal treatment? A systematic review and meta-analysis. J Periodontal Res. Department of Stomatology, Division of Periodontics, Faculty of Dentistry, University of São Paulo, São Paulo, Brazil, United States: Blackwell Munksgaard; 2018;53:12–21. |
| 20. Amghar-Maach S, Gay-Escoda C, Sánchez-Garcés MÁ. Regeneration of periodontal bone defects with dental pulp stem cells grafting: Systematic Review. J Clin Exp Dent. 2019;11:e373–81. |
| 21. Anandakumar S, Malaiappan S. Effect of Subgingival Irrigation with Natural Products as an Adjunct to Scaling and Root Planing in the Treatment of Chronic Periodontitis- A Systematic Review. J Clin DIAGNOSTIC Res. 2018;12:ZC17–20. |
| 22. Andreotti AM, Goiato MC, Nobrega AS, Freitas Da Silva E V, Filho HG, Pellizzer EP, et al. Relationship between implant stability measurements obtained by two different devices: A systematic review. J Periodontol. 2017;88:281–8. |
| 23. Angaji M, Gelskey S, Nogueira-Filho G, Brothwell D. A systematic review of clinical efficacy of adjunctive antibiotics in the treatment of smokers with periodontitis. J Periodontol. United States; 2010;81:1518–28. |
| 24. Angelillo IF, Nobile CGA, Pavia M. Evaluation of the effectiveness of a pre-brushing rinse in plaque removal: A meta-analysis. J Clin Periodontol. Department of Hygiene, Medical School, University of Catanzaro Magna Græcia, Catanzaro, Italy; 2002;29:301–9. |
| 25. Angst PDM, Stadler AF, Oppermann RV, Gomes SC. Microbiological outcomes from different periodontal maintenance interventions: a systematic review. Braz. Oral Res. 2017. p. e33. |
| 26. Annibali S, Bignozzi I, Cristalli MP, Graziani F, La Monaca G, Polimeni A. Peri-implant marginal bone level: A systematic review and meta-analysis of studies comparing platform switching versus conventionally restored implants. J Clin Periodontol. 2012;39:1097–113. |
| 27. Annunziata M, Nastri L, Cecoro G, Guida L. The Use of Poly-d,l-lactic Acid (PDLLA) Devices for Bone Augmentation Techniques: A Systematic Review. Molecules. Switzerland; 2017;22. |
| 28. Arnold S, Koletsi D, Patcas R, Eliades T. The effect of bracket ligation on the periodontal status of adolescents undergoing orthodontic treatment. A systematic review and meta-analysis. J Dent. England; 2016;54:13–24. |
| 29. ASSEM NZ, ALVES MLF, LOPES AB, GUALBERTO JUNIOR EC, GARCIA VG, THEODORO LH. Antibiotic therapy as an adjunct to scaling and root planing in smokers: a systematic review and meta-analysis. Braz Oral Res. 2017;31:e67. |
| 30. Atieh MA, Alsabeeha N, Tawse-Smith A, Payne AG. Xenogeneic collagen matrix for periodontal plastic surgery procedures: a systematic review and meta-analysis. J Periodontal Res. 2016;51:438–52. |
| 31. Avila-Ortiz G, Chambrone L, Vignoletti F. Effect of alveolar ridge preservation interventions following tooth extraction: A systematic review and meta-analysis. J Clin Periodontol. 2019;46:195–223. |

Additional file 3. continued

| 32. Azarpazhooh A, Shah PS, Tenenbaum HC, Goldberg MB. The effect of photodynamic therapy for periodontitis: A systematic review and meta-analysis. J Periodontol. Community Dental Health Services Research Unit, Faculty of Dentistry, University of Toronto, 124 Edward St., Toronto, ON M5G 1G6, United States; 2010;81:4–14. |
| --- |
| 33. Bae J-H, Kim Y-K, Myung S-K. Desensitizing toothpaste versus placebo for dentin hypersensitivity: A systematic review and meta-analysis. J Clin Periodontol. 2015;42:131–41. |
| 34. Bae J-H, Kim Y-K, Myung S-K. Effects of platelet-rich plasma on sinus bone graft: Meta-analysis. J Periodontol. 2011;82:660–7. |
| 35. Baeza M, Morales A, Cisterna C, Cavalla F, Jara G, Isamitt Y, et al. Effect of periodontal treatment in patients with periodontitis and diabetes: systematic review and meta-analysis. J Appl Oral Sci. Brazil; 2020;28:e20190248. |
| 36. Barbato L, Kalemaj Z, Buti J, Baccini M, La Marca M, Duvina M, et al. Effect of surgical intervention for removal of mandibular third molar on periodontal healing of adjacent mandibular second molar: A systematic review and Bayesian network meta-analysis. J Periodontol. 2016;87:291–302. |
| 37. Bassetti MAt alveolar ridge splitting/expansion technique: a systematic review., Bassetti RG, Bosshardt DD. The alveolar ridge splitting/expansion technique: a systematic review. Clin Oral Implants Res. Denmark; 2016;27:310–24. |
| 38. Bassetti RG, Stahli A, Bassetti MA, Sculean A. Soft tissue augmentation procedures at second-stage surgery: a systematic review. Clin Oral Investig. Germany; 2016;21:1369–87. |
| 39. Bassir SH, El Kholy K, Chen C-Y, Lee KH, Intini G. Outcome of early dental implant placement versus other dental implant placement protocols: A systematic review and meta-analysis. J Periodontol. 2019;90:493–506. |
| 40. Behdin S, Monje A, Lin G-H, Edwards B, Othman A, Wang H-L. Effectiveness of Laser Application for Periodontal Surgical Therapy: Systematic Review and Meta-Analysis. J Periodontol. United States; 2015;86:1352–63. |
| 41. Berchier CE, Slot DE, Haps S, Van der Weijden GA. The efficacy of dental floss in addition to a toothbrush on plaque and parameters of gingival inflammation: a systematic review. Int J Dent Hyg. England; 2008;6:265–79. |
| 42. Berchier CE, Slot DE, Van der Weijden GA. The efficacy of 0.12% chlorhexidine mouthrinse compared with 0.2% on plaque accumulation and periodontal parameters: a systematic review. J Clin Periodontol. United States; 2010;37:829–39. |
| 43. Bertl K, Bruckmann C, Isberg P-E, Klinge B, Gotfredsen K, Stavropoulos A. Hyaluronan in non-surgical and surgical periodontal therapy: A systematic review. J Clin Periodontol. 2015;42:236–46. |
| 44. Bertl K, Steiner I, Pandis N, Buhlin K, Klinge B, Stavropoulos A. Statins in nonsurgical and surgical periodontal therapy. A systematic review and meta-analysis of preclinical in vivo trials. J Periodontal Res. 2018;53:267–87. |
| 45. Bertl K, Melchard M, Pandis N, Muller-Kern M, Stavropoulos A. Soft tissue substitutes in non-root coverage procedures: a systematic review and  meta-analysis. Clin Oral Investig. Germany; 2017;21:505–18. |
| 46. Bertl K, Parllaku A, Pandis N, Buhlin K, Klinge B, Stavropoulos A. The effect of local and systemic statin use as an adjunct to non-surgical and surgical periodontal therapy-A systematic review and meta-analysis. J Dent. England; 2017;67:18–28. |
| 47. Biesbrock A, He T, DiGennaro J, Zou Y, Ramsey D, Garcia-Godoy F. The effects of bioavailable gluconate chelated stannous fluoride dentifrice on gingival bleeding: Meta-analysis of eighteen randomized controlled trials. J Clin Periodontol. United States; 2019;46:1205–16. |

Additional file 3. continued

| 48. Bonito AJ, Lux L, Lohr KN. Impact of local adjuncts to scaling and root planing in periodontal disease therapy: a systematic review. J Periodontol. United States; 2005;76:1227–36. |
| --- |
| 49. Bono A, Brunotto M. Amoxicillin/metronidazole or scaling and root planing in the treatment of chronic periodontitis. Acta Odontol Latinoam. Argentina; 2010;23:196–203. |
| 50. Bouziane A, Ahid S, Abouqal R, Ennibi O. Effect of periodontal therapy on prevention of gastric Helicobacter pylori recurrence: A systematic review and meta-analysis. J Clin Periodontol. 2012;39:1166–73. |
| 51. Bright R, Hynes K, Gronthos S, Bartold PM. Periodontal ligament-derived cells for periodontal regeneration in animal models: a systematic review. J Periodontal Res. United States; 2015;50:160–72. |
| 52. Buset SL, Zitzmann NU, Weiger R, Walter C. Non-surgical periodontal therapy supplemented with systemically administered azithromycin: a systematic review of RCTs. Clin Oral Investig. 2015;19:1763–75. |
| 53. Buti J, Baccini M, Nieri M, La Marca M, Pini-Prato GP. Bayesian network meta-analysis of root coverage procedures: Ranking efficacy and identification of best treatment. J Clin Periodontol. 2013;40:372–86. |
| 54. Caballe-Serrano J, Abdeslam-Mohamed Y, Munar-Frau A, Fujioka-Kobayashi M, Hernandez-Alfaro F, Miron R, et al. Adsorption and release kinetics of growth factors on barrier membranes for guided tissue/bone regeneration: A systematic review. Arch Oral Biol. England; 2019;100:57–68. |
| 55. Cairo F, Nieri M, Pagliaro U. Efficacy of periodontal plastic surgery procedures in the treatment of localized facial gingival recessions. A systematic review. J Clin Periodontol. 2014;41:S44–62. |
| 56. Cairo F, Pagliaro U, Nieri M. Treatment of gingival recession with coronally advanced flap procedures: A systematic review. J Clin Periodontol. 2008;35:136–62. |
| 57. Camps-Font O, Burgueño-Barris G, Figueiredo R, Jung RE, Gay-Escoda C, Valmaseda-Castellón E. Interventions for Dental Implant Placement in Atrophic Edentulous Mandibles: Vertical Bone Augmentation and Alternative Treatments. A Meta-Analysis of Randomized Clinical Trials. J Periodontol. 2016;87:1444–57. |
| 58. Cao R, Li Q, Chen Y, Yao M, Wu Q, Zhou H. Efficacy of locally-delivered statins adjunct to non-surgical periodontal therapy for chronic periodontitis: A Bayesian network analysis. BMC Oral Health. Department of Prosthodontics, Xiangya Stomatological Hospital, School of Stomatology, Central South University, 72 Xiangya Road, Changsha, 410000, China: BioMed Central Ltd.; 2019;19. |
| 59. Cao R, Li Q, Wu Q, Yao M, Chen Y, Zhou H. Effect of non-surgical periodontal therapy on glycemic control of type 2 diabetes mellitus: A systematic review and Bayesian network meta-analysis [Internet]. BMC Oral Health. 2019. p. 176. |
| 60. Castro MML, Duarte NN, Nascimento PC, Magno MB, Fagundes NCF, Flores-Mir C, et al. Antioxidants as Adjuvants in Periodontitis Treatment: A Systematic Review and Meta-Analysis. Oxid Med Cell Longev. United States; 2019;2019:9187978. |
| 61. Chambrone L, Chambrone LA, Lima LA. Effects of occlusal overload on peri-implant tissue health: A systematic review of animal-model studies. J Periodontol. 2010;81:1367–78. |
| 62. Chambrone L, Foz AM, Guglielmetti MR, Pannuti CM, Artese HPC, Feres M, et al. Periodontitis and chronic kidney disease: A systematic review of the association of diseases and the effect of periodontal treatment on estimated glomerular filtration rate. J Clin Periodontol. 2013;40:443–56. |
| 63. Chambrone L, Preshaw PM, Rosa EF, Heasman PA, Romito GA, Pannuti CM, et al. Effects of smoking cessation on the outcomes of non-surgical periodontal therapy: A systematic review and individual patient data meta-analysis. J Clin Periodontol. 2013;40:607–15. |

Additional file 3. continued

| 64. Chambrone L, Tatakis DN. Long-Term outcomes of untreated buccal gingival recessions: A systematic review and meta-Analysis. J Periodontol. Unit of Basic Oral Investigation, School of Dentistry, El Bosque University, Bogota, Colombia: American Academy of Periodontology; 2016;87:796–808. |
| --- |
| 65. Chambrone L, Lima LA, Pustiglioni FE, Chambrone LA. Systematic review of periodontal plastic surgery in the treatment of multiple recession-type defects. J Can Dent Assoc. Canada; 2009;75:203a-203f. |
| 66. Chambrone L, Ramos UD, Reynolds MA. Infrared lasers for the treatment of moderate to severe periodontitis: An American Academy of Periodontology best evidence review. J Periodontol. United States; 2018;89:743–65. |
| 67. Chambrone L, Vargas M, Arboleda S, Serna M, Guerrero M, De Sousa J, et al. Efficacy of Local and Systemic Antimicrobials in the Non-Surgical Treatment of Smokers With Chronic Periodontitis: A Systematic Review. J Periodontol. United States; 2016;87:1320–32. |
| 68. Chambrone L, Wang H-L, Romanos GE. Antimicrobial photodynamic therapy for the treatment of periodontitis and peri-implantitis: An American Academy of Periodontology best evidence review. J Periodontol. United States; 2018;89:783–803. |
| 69. Chen Y, Wong RWK, McGrath C, Hagg U, Seneviratne CJ. Natural compounds containing mouthrinses in the management of dental plaque and gingivitis: A systematic review. Clin Oral Investig. Department of Orthodontics, Affiliated Stomatological Hospital of Fujian Medical University, Fujian, China: Springer Verlag; 2014;18:1–16. |
| 70. Chen Z, Zhang Y, Li J, Wang H-L, Yu H. Influence of Laser-Microtextured Surface Collar on Marginal Bone Loss and Peri-Implant Soft Tissue Response: A Systematic Review and Meta-Analysis. J Periodontol. 2017;88:651–62. |
| 71. Clauser C, Nieri M, Franceschi D, Pagliaro U, Pini-Prato G. Evidence-based mucogingival therapy. Part 2: Ordinary and individual patient data meta-analyses of surgical treatment of recession using complete root coverage as the outcome variable. J Periodontol. United States; 2003;74:741–56. |
| 72. Clementini M, Morlupi A, Canullo L, Agrestini C, Barlattani A. Success rate of dental implants inserted in horizontal and vertical guided bone regenerated areas: a systematic review. Int J Oral Maxillofac Surg. Denmark; 2012;41:847–52. |
| 73. Clementini M, Tiravia L, De Risi V, Vittorini Orgeas G, Mannocci A, De Sanctis M. Dimensional changes after immediate implant placement with or without simultaneous regenerative procedures: A systematic review and meta-analysis. J Clin Periodontol. 2015;42:666–77. |
| 74. Cosyn J, De Lat L, Seyssens L, Doornewaard R, Deschepper E, Vervaeke S. The effectiveness of immediate implant placement for single tooth replacement compared to delayed implant placement: A systematic review and meta-analysis. J Clin Periodontol. 2019;46:224–41. |
| 75. Cosyn J, Sabzevar MM. A Systematic Review on the Effects of Subgingival Chlorhexidine Gel Administration in the Treatment of Chronic Periodontitis. J Periodontol. Department of Periodontology, School of Dental Medicine, Free University of Brussels, Laarbeeklaan 103, B-1090 Brussels, Belgium; 2005;76:1805–13. |
| 76. Cosyn J, Wyn I. A systematic review on the effects of the chlorhexidine chip when used as an adjunct to scaling and root planing in the treatment of chronic periodontitis. J Periodontol. United States; 2006;77:257–64. |
| 77. da Costa LFNP, Amaral C da SF, Barbirato D da S, Leao ATT, Fogacci MF. Chlorhexidine mouthwash as an adjunct to mechanical therapy in chronic periodontitis: A meta-analysis. J Am Dent Assoc. England; 2017;148:308–18. |

Additional file 3. continued

| 78. da Rosa Moreira Bastos RT, Blagitz MN, de Castro Aragón MLS, Maia LC, Normando D. Periodontal side effects of rapid and slow maxillary expansion: A systematic review. Angle Orthod. 2019;89:651–60. |
| --- |
| 79. da Silva JC, Muniz FWMG, Oballe HJR, Andrades M, Rosing CK, Cavagni J. The effect of periodontal therapy on oxidative stress biomarkers: A systematic review. J Clin Periodontol. United States; 2018;45:1222–37. |
| 80. Dai A, Huang J-P, Ding P-H, Chen L-L. Long-term stability of root coverage procedures for single gingival recessions: A systematic review and meta-analysis. J Clin Periodontol. 2019;46:572–85. |
| 81. Danesh-Sani SA, Engebretson SP, Janal MN. Histomorphometric results of different grafting materials and effect of healing time on bone maturation after sinus floor augmentation: a systematic review and meta-analysis. J Periodontal Res. 2017;52:301–12. |
| 82. Darby IB, Morris KH. A systematic review of the use of growth factors in human periodontal regeneration. J Periodontol. United States; 2013;84:465–76. |
| 83. Davies RM, Ellwood RP, Davies GM. The effectiveness of a toothpaste containing triclosan and polyvinyl-methyl ether maleic acid copolymer in improving plaque control and gingival health: a systematic review. J Clin Periodontol. United States; 2004;31:1029–33. |
| 84. de Almeida JM, Matheus HR, Rodrigues Gusman DJ, Faleiros PL, Januario de Araujo N, Noronha Novaes VC. Effectiveness of Mechanical Debridement Combined With Adjunctive Therapies for Nonsurgical Treatment of Periimplantitis: A Systematic Review. Implant Dent. United States; 2017;26:137–44. |
| 85. de Brandao ML, Vettore M V, Vidigal Junior GM, De Brandão ML, Vettore M V, Vidigal Júnior GM. Peri-implant bone loss in cement- and screw-retained prostheses: systematic review and meta-analysis. J Clin Periodontol. United States; 2013;40:287–95. |
| 86. de Camargo L, da Silva SN, Chambrone L. Efficacy of toothbrushing procedures performed in intensive care units in reducing the risk of ventilator-associated pneumonia: A systematic review. J Periodontal Res. United States; 2019;54:601–11. |
| 87. Del Fabbro M, Bortolin M, Taschieri S, Weinstein R. Is platelet concentrate advantageous for the surgical treatment of periodontal diseases? A systematic review and meta-analysis. J Periodontol. United States; 2011;82:1100–11. |
| 88. Deutscher H, Derman S, Barbe AG, Seemann R, Noack MJ. The effect of professional tooth cleaning or non-surgical periodontal therapy on  oral halitosis in patients with periodontal diseases. A systematic review. Int J Dent Hyg. England; 2018;16:36–47. |
| 89. Dhingra K, Vandana KL. Prophylactic vaccination against periodontal disease: A systematic review of preclinical studies. J Periodontol. 2010;81:1529–46. |
| 90. Donos N, Mardas N, Chadha V. Clinical outcomes of implants following lateral bone augmentation: systematic assessment of available options (barrier membranes, bone grafts, split osteotomy). J Clin Periodontol. United States; 2008;35:173–202. |
| 91. Dragan IF, Hotlzman LP, Karimbux NY, Morin RA, Bassir SH. Clinical Outcomes of Comparing Soft Tissue Alternatives to Free Gingival Graft: A Systematic Review and Meta-Analysis. J Evid Based Dent Pract. United States; 2017;17:370-380.e3. |
| 92. Eberhard J, Jervoe-Storm P-M, Needleman I, Worthington H, Jepsen S. Full-mouth treatment concepts for chronic periodontitis: a systematic review. J Clin Periodontol. United States; 2008;35:591–604. |
| 93. Eliezer M, Imber J-C, Sculean A, Pandis N, Teich S. Hyaluronic acid as adjunctive to non-surgical and surgical periodontal therapy: a systematic review and meta-analysis. Clin Oral Investig. Germany; 2019;23:3423–35. |

Additional file 3. continued

| 94. Elkerbout TA, Slot DE, Rosema NAM, Van der Weijden GA. How effective is a powered toothbrush as compared to a manual toothbrush? A systematic review and meta-analysis of single brushing exercises. Int J Dent Hyg. England; 2020;18:17–26. |
| --- |
| 95. Engebretson S, Kocher T. Evidence that periodontal treatment improves diabetes outcomes: A systematic review and meta-analysis. J Periodontol. 2013;84:S153–63. |
| 96. Escribano M, Figuero E, Martin C, Tobias A, Serrano J, Roldan S, et al. Efficacy of adjunctive anti-plaque chemical agents: a systematic review and network meta-analyses of the Turesky modification of the Quigley and Hein plaque index. J Clin Periodontol. United States; 2016;43:1059–73. |
| 97. Eshghipour B, Tofighi H, Nehal F, Vohra F, Javed F, Akram Z. Effect of scaling and root planing on gingival crevicular fluid cytokine/chemokine levels in smokers with chronic periodontitis: A systematic review. J Investig Clin Dent. Australia; 2018;9:e12327. |
| 98. Fang H, Han M, Li QL, Cao CY, Xia R, Zhang ZH. Comparison of full-mouth disinfection and quadrant-wise scaling in the treatment of adult chronic periodontitis: a systematic review and meta-analysis. J. Periodontal Res. 2016. p. 417–30. |
| 99. Farman M, Joshi RI. Full-mouth treatment versus quadrant root surface debridement in the treatment of chronic periodontitis: a systematic review. Br Dent J. England; 2008;205:E18; discussion 496-7. |
| 100. Figuero E, Nóbrega DF, García-Gargallo M, Tenuta LMA, Herrera D, Carvalho JC. Mechanical and chemical plaque control in the simultaneous management of gingivitis and caries: a systematic review. J Clin Periodontol. 2017;44:S116–34. |
| 101. Figuero E, Herrera D, Tobías A, Serrano J, Roldán S, Escribano M, et al. Efficacy of adjunctive anti-plaque chemical agents in managing gingivitis: A systematic review and network meta-analyses. J Clin Periodontol. 2019;46:723–39. |
| 102. Foz AM, Artese HPC, Horliana ACRT, Pannuti CM, Romito GA. Occlusal adjustment associated with periodontal therapy - A systematic review. J Dent. 2012;40:1025–35. |
| 103. Fritoli A, Goncalves C, Faveri M, Figueiredo LC, Perez-Chaparro PJ, Fermiano D, et al. The effect of systemic antibiotics administered during the active phase of non-surgical periodontal therapy or after the healing phase: a systematic review. J Appl Oral Sci. Brazil; 2015;23:249–54. |
| 104. Gapski R, Parks CA, Wang H-L. Acellular Dermal Matrix for Mucogingival Surgery: A Meta-Analysis. J Periodontol. 2005;76:1814–22. |
| 105. Garcia Canas P, Khouly I, Sanz J, Loomer PM, Canas PG, Khouly I, et al. Effectiveness of systemic antimicrobial therapy in combination with scaling and root planing in the treatment of periodontitis: A systematic review. J Am Dent Assoc. Department of Periodontology and Implant Dentistry, College of Dentistry, New York University, 421 First Ave., 3W, New York, NY, United States, England: American Dental Association; 2015;146:150–63. |
| 106. Gartenmann SJ, Weydlich Y V, Steppacher SL, Heumann C, Attin T, Schmidlin PR. The effect of green tea as an adjunct to scaling and root planing in non-surgical periodontitis therapy: a systematic review. Clin Oral Investig. Germany; 2019;23:1–20. |
| 107. Gbinigie O, Onakpoya I, Spencer E, McCall MacBain M, Heneghan C. Effect of oil pulling in promoting oro dental hygiene: A systematic review of randomized clinical trials. Complement Ther Med. Scotland; 2016;26:47–54. |
| 108. Graziani F, Gennai S, Roldán S, Discepoli N, Buti J, Madianos P, et al. Efficacy of periodontal plastic procedures in the treatment of multiple gingival recessions. J Clin Periodontol. 2014;41:S63–76. |
| 109. Grellmann AP, Sfreddo CS, Maier J, Lenzi TL, Zanatta FB. Systemic antimicrobials adjuvant to periodontal therapy in diabetic subjects: A meta-analysis. J. Clin. Periodontol. 2016. p. 250–60. |

Additional file 3. continued

| 110. Grender J, Adam R, Zou Y. The effects of oscillating-rotating electric toothbrushes on plaque and gingival  health: A meta-analysis. Am J Dent. United States; 2020;33:3–11. |
| --- |
| 111. Haas AN, Wagner TP, Muniz FWMG, Fiorini T, Cavagni J, Celeste RK. Essential oils-containing mouthwashes for gingivitis and plaque: Meta-analyses and meta-regression. J Dent. 2016;55:7–15. |
| 112. Haffajee AD, Socransky SS, Gunsolley JC. Systemic anti-infective periodontal therapy. A systematic review. Ann Periodontol. United States; 2003;8:115–81. |
| 113. Hallmon WW, Rees TD. Local anti-infective therapy: mechanical and physical approaches. A systematic review. Ann Periodontol. United States; 2003;8:99–114. |
| 114. Hanes PJ, Purvis JP. Local anti-infective therapy: pharmacological agents. A systematic review. Ann Periodontol. United States; 2003;8:79–98. |
| 115. Haps S, Slot DE, Berchier CE, Van der Weijden GA. The effect of cetylpyridinium chloride-containing mouth rinses as adjuncts to toothbrushing on plaque and parameters of gingival inflammation: a systematic review. Int J Dent Hyg. England; 2008;6:290–303. |
| 116. Heasman PA, McCracken GI, Steen N. Supportive periodontal care: The effect of periodic subgingival debridement compared with supragingival prophylaxis with respect to clinical outcomes. J Clin Periodontol. School of Dental Science, University of Newcastle upon Tyne, Newcastle upon Tyne, United Kingdom; 2002;29:163–72. |
| 117. Heitz-Mayfield LJA, Trombelli L, Heitz F, Needleman I, Moles D. A systematic review of the effect of surgical debridement vs non-surgical debridement for the treatment of chronic periodontitis. J Clin Periodontol. United States; 2002;29 Suppl 3:92. |
| 118. Herrera D, Sanz M, Jepsen S, Needleman I, Roldan S. A systematic review on the effect of systemic antimicrobials as an adjunct to scaling and root planing in periodontitis patients. J Clin Periodontol. United States; 2002;29 Suppl 3:132–6. |
| 119. Hoenderdos NL, Slot DE, Paraskevas S, Van der Weijden GA. The efficacy of woodsticks on plaque and gingival inflammation: a systematic review. Int J Dent Hyg. England; 2008;6:280–9. |
| 120. Hoogteijling F, Hennequin-Hoenderdos NL, Van der Weijden GA, Slot DE. The effect of tapered toothbrush filaments compared to end-rounded filaments on dental plaque, gingivitis and gingival abrasion: a systematic review and meta-analysis. Int J Dent Hyg. England; 2018;16:3–12. |
| 121. Hossainian N, Slot DE, Afennich F, Van der Weijden GA. The effects of hydrogen peroxide mouthwashes on the prevention of plaque and gingival inflammation: a systematic review. Int J Dent Hyg. England; 2011;9:171–81. |
| 122. Hou X, Yuan J, Aisaiti A, Liu Y, Zhao J. The effect of platelet-rich plasma on clinical outcomes of the surgical treatment of periodontal intrabony defects: A systematic review and meta-analysis. BMC Oral Health. England; 2016;16:71. |
| 123. Hung H-C, Douglass CW. Meta-analysis of the effect of scaling and root planing, surgical treatment and antibiotic therapies on periodontal probing depth and attachment loss. J Clin Periodontol. Harvard School of Public Health, Boston, MA, United States; 2002;29:975–86. |
| 124. Ikram S, Hassan N, Raffat MA, Mirza S, Akram Z. Systematic review and meta-analysis of double-blind, placebo-controlled, randomized clinical trials using probiotics in chronic periodontitis. J Investig Clin Dent. Australia; 2018;9:e12338. |
| 125. Incerti-Parenti S, Checchi V, Ippolito DR, Gracco A, Alessandri-Bonetti G. Periodontal status after surgical-orthodontic treatment of labially impacted canines with different surgical techniques: A systematic review. Am J Orthod Dentofacial Orthop. United States; 2016;149:463–72. |
| 126. Iocca O, Farcomeni A, Pardiñas Lopez S, Talib HS. Alveolar ridge preservation after tooth extraction: a Bayesian Network meta-analysis of grafting materials efficacy on prevention of bone height and width reduction. J Clin Periodontol. 2017;44:104–14. |

Additional file 3. continued

| 127. Jagannathan N, Acharya A, Yi Farn O, Li KYY, Nibali L, Pelekos G. Disease severity, debridement approach and timing of drug modify outcomes of adjunctive azithromycin in non-surgical management of chronic periodontitis: A multivariate meta-analysis. BMC Oral Health. Periodontology, Faculty of Dentistry, University of Hong Kong, Prince Philip Dental Hospital, 34 Hospital Road, Sai Yin Pun, Hong Kong: BioMed Central Ltd.; 2019;19. |
| --- |
| 128. Jain A, Gupta J, Bansal D, Sood S, Gupta S, Jain A. Effect of scaling and root planing as monotherapy on glycemic control in patients of Type 2 diabetes with chronic periodontitis: A systematic review and meta-analysis. J Indian Soc Periodontol. Department of Periodontics, Dr. Harvansh Singh Judge Institute of Dental Sciences and Hospital, Panjab University, Sector-25, Chandigarh, Punjab  160 014, India: Wolters Kluwer Medknow Publications; 2019;23:303–10. |
| 129. Javed F, Salehpoor D, Al-Dhafeeri T, Yousuf M, Malmstrom H, Khan J, et al. Is adjunctive photodynamic therapy more effective than scaling and root planing alone in the treatment of periodontal disease in hyperglycemic patients? A systematic review. Photodiagnosis Photodyn Ther. Netherlands; 2018;22:1–6. |
| 130. Jepsen S, Eberhard J, Herrera D, Needleman I. A systematic review of guided tissue regeneration for periodontal furcation defects. What is the effect of guided tissue regeneration compared with surgical debridement in the treatment of furcation defects? J Clin Periodontol. United States; 2002;29 Suppl 3:102–3. |
| 131. Joseph B, Janam P, Narayanan S, Anil S. Is Antimicrobial Photodynamic Therapy Effective as an Adjunct to Scaling and Root Planing in Patients with Chronic Periodontitis? A Systematic Review. Biomolecules. Switzerland; 2017;7. |
| 132. Jung RE, Thoma DS, Hammerle CHF. Assessment of the potential of growth factors for localized alveolar ridge augmentation: a systematic review. J Clin Periodontol. 2008;35:255–81. |
| 133. Karlsson MR, Diogo Lofgren CI, Jansson HM, Diogo Löfgren CI, Jansson HM. The effect of laser therapy as an adjunct to non-surgical periodontal treatment in subjects with chronic periodontitis: a systematic review. J Periodontol. United States; 2008;79:2021–8. |
| 134. Katsamakis S, Slot DE, Van Der Sluis LWM, Van Der Weijden F. Histological responses of the periodontium to MTA: A systematic review. J Clin Periodontol. 2013;40:334–44. |
| 135. Keestra JAJ, Grosjean I, Coucke W, Quirynen M, Teughels W. Non-surgical periodontal therapy with systemic antibiotics in patients with untreated aggressive periodontitis: A systematic review and meta-analysis. J Periodontal Res. Department of Oral Health Sciences, Periodontology, KU Leuven and University of Leuven, Leuven, Belgium, United States: Blackwell Munksgaard; 2015;50:689–706. |
| 136. Kellesarian SV, Malignaggi VR, Majoka HA, Al-Kheraif AA, Kellesarian TV, Romanos GE, et al. Effect of laser-assisted scaling and root planing on the expression of pro-inflammatory cytokines in the gingival crevicular fluid of patients with chronic periodontitis: A systematic review. Photodiagnosis Photodyn Ther. 2017;18:63–77. |
| 137. Keukenmeester RS, Slot DE, Putt MS, Van der Weijden GA. The effect of medicated, sugar-free chewing gum on plaque and clinical parameters of gingival inflammation: a systematic review. Int J Dent Hyg. England; 2014;12:2–16. |
| 138. Khoshkam V, Chan HL, Lin GH, MacEachern MP, Monje A, Suarez F, et al. Reconstructive procedures for treating peri-implantitis: a systematic review. J Dent Res. United States; 2013;92:131S-8S. |
| 139. Kim AJ, Lo AJ, Pullin DA, Thornton-Johnson DS, Karimbux NY. Scaling and root planing treatment for periodontitis to reduce preterm birth and  low birth weight: a systematic review and meta-analysis of randomized controlled trials. J Periodontol. United States; 2012;83:1508–19. |
| 140. Kinaia BM, Steiger J, Neely AL, Shah M, Bhola M. Treatment of Class II molar furcation involvement: meta-analyses of reentry results. J Periodontol. United States; 2011;82:413–28. |

Additional file 3. continued

| 141. Kotsakis GA, Konstantinidis I, Karoussis IK, Ma X, Chu H. Systematic review and meta-analysis of the effect of various laser wavelengths in the treatment of peri-implantitis. J Periodontol. 2014;85:1203–13. |
| --- |
| 142. Kotsovilis S, Karoussis IK, Trianti M, Fourmousis I. Therapy of peri-implantitis: A systematic review: Review article. J Clin Periodontol. Department of Periodontology, School of Dentistry, University of Athens, Athens, Greece; 2008;35:621–9. |
| 143. Kotsovilis S, Markou N, Pepelassi E, Nikolidakis D. The adjunctive use of platelet-rich plasma in the therapy of periodontal intraosseous defects: A systematic review. J Periodontal Res. 2010;45:428–43. |
| 144. Kumar S, Madurantakam P. Limited evidence shows short-term benefit of probiotics when used as an adjunct to scaling and root planing in the treatment of chronic periodontitis. Evid Based Dent. United States; 2017;18:109–10. |
| 145. Lang NP, Tan WC, Krahenmann MA, Zwahlen M. A systematic review of the effects of full-mouth debridement with and without antiseptics in patients with chronic periodontitis. J Clin Periodontol. United States; 2008;35:8–21. |
| 146. Laugisch O, Cosgarea R, Nikou G, Nikolidakis D, Donos N, Salvi GE, et al. Histologic evidence of periodontal regeneration in furcation defects: a systematic review. Clin Oral Investig. Germany; 2019;23:2861–906. |
| 147. Li A, Yang H, Zhang J, Chen S, Wang H, Gao Y. Additive effectiveness of autologous platelet-rich fibrin in the treatment of intrabony defects: A PRISMA-compliant meta-analysis. Medicine (Baltimore). United States; 2019;98:e14759. |
| 148. Li F, Jiang P, Pan J, Liu C, Zheng L. Synergistic Application of Platelet-Rich Fibrin and 1% Alendronate in Periodontal Bone Regeneration: A Meta-Analysis. Biomed Res Int. United States; 2019;2019:9148183. |
| 149. Li W, Xiao L, Hu J. The use of enamel matrix derivative alone versus in combination with bone grafts  to treat patients with periodontal intrabony defects: a meta-analysis. J Am Dent Assoc. England; 2012;143:e46-56. |
| 150. Lin G-H, Chan H-L, Wang H-L. Effects of Currently Available Surgical and Restorative Interventions on Reducing Midfacial Mucosal Recession of Immediately Placed Single-Tooth Implants: A Systematic Review. J Periodontol. 2014;85:92–102. |
| 151. Lin P-Y, Cheng Y-W, Chu C-Y, Chien K-L, Lin C-P, Tu Y-K. In-office treatment for dentin hypersensitivity: A systematic review and network meta-analysis. J Clin Periodontol. 2013;40:53–64. |
| 152. Lira Junior R, Santos C de MM, Oliveira BH, Fischer RG, Santos APP. Effects on HbA1c in diabetic patients of adjunctive use of systemic antibiotics in nonsurgical periodontal treatment: A systematic review. J Dent. 2017;66:1–7. |
| 153. Liu Y, Hu B, Zhou J, Li W, Liu Q, Song J. The Effect of Enamel Matrix Derivative Alone Versus in Combination with Alloplastic Materials to Treat Intrabony Defects: A Meta-analysis. Int J Periodontics Restorative Dent. United States; 2017;37:e224–33. |
| 154. Ma L, Zhang X, Ma Z, Shi H, Zhang Y, Wu M, et al. Clinical Effectiveness of Er: YAG Lasers Adjunct to Scaling and Root Planing in Non-Surgical Treatment of Chronic Periodontitis: A Meta-Analysis of Randomized Controlled Trials. Med Sci Monit. United States; 2018;24:7090–9. |
| 155. Mailoa J, Lin G-H, Chan H-L, MacEachern M, Wang H-L. Clinical outcomes of using lasers for peri-implantitis surface detoxification: A systematic review and meta-analysis. J Periodontol. 2014;85:1194–202. |
| 156. Mailoa J, Lin G-H, Khoshkam V, MacEachern M, Chan H-L, Wang H-L. Long-Term Effect of Four Surgical Periodontal Therapies and One Non-Surgical Therapy: A Systematic Review and Meta-Analysis. J Periodontol. United States; 2015;86:1150–8. |

Additional file 3. continued

| 157. Matesanz-Pérez P, García-Gargallo M, Figuero E, Bascones-Martínez A, Sanz M, Herrera D, et al. A systematic review on the effects of local antimicrobials as adjuncts to subgingival debridement, compared with subgingival debridement alone, in the treatment of chronic periodontitis. J Clin Periodontol. Graduate Periodontology, Faculty of Odontology, University Complutense, Madrid, Spain, United States; 2013;40:227–41. |
| --- |
| 158. Matsubara VH, Bandara HMHN, Ishikawa KH, Mayer MPA, Samaranayake LP. The role of probiotic bacteria in managing periodontal disease: a systematic review. Expert Rev Anti Infect Ther. 2016;14:643–55. |
| 159. Matthews D. Local antimicrobials in addition to scaling and root planing provide statistically significant but not clinically important benefit. Evid Based Dent. England; 2013;14:87–8. |
| 160. McGowan K, McGowan T, Ivanovski S. Optimal dose and duration of amoxicillin-plus-metronidazole as an adjunct to non-surgical periodontal therapy: A systematic review and meta-analysis of randomized, placebo-controlled trials. J Clin Periodontol. 2018;45:56–67. |
| 161. McLaughlin M, Duane B. Evidence that full-mouth scaling superior to conventional treatment approaches is unclear. Evid Based Dent. England; 2016;17:23–4. |
| 162. Meza-Mauricio J, Soto-Penaloza D, Penarrocha-Oltra D, Montiel-Company JM, Peruzzo DC. Locally applied statins as adjuvants to non-surgical periodontal treatment for chronic periodontitis: a systematic review and meta-analysis. Clin Oral Investig. Germany; 2018;22:2413–30. |
| 163. Migliorati M, Isaia L, Cassaro A, Rivetti A, Silvestrini-Biavati F, Gastaldo L, et al. Efficacy of professional hygiene and prophylaxis on preventing plaque increase in orthodontic patients with multibracket appliances: a systematic review. Eur J Orthod. England; 2015;37:297–307. |
| 164. Mokeem S. Efficacy of adjunctive low-level laser therapy in the treatment of aggressive periodontitis: A systematic review. J Investig Clin Dent. Australia; 2018;9:e12361. |
| 165. Monje A, Kramp AR, Criado E, Suarez-Lopez Del Amo F, Garaicoa-Pazmino C, Gargallo-Albiol J, et al. Effect of periodontal dressing on non-surgical periodontal treatment outcomes: a  systematic review. Int J Dent Hyg. England; 2016;14:161–7. |
| 166. Moro MG, Silveira Souto ML, Franco GCN, Holzhausen M, Pannuti CM. Efficacy of local phytotherapy in the nonsurgical treatment of periodontal disease: A systematic review. J Periodontal Res. Department of Stomatology, Division of Periodontics, School of Dentistry, University of São Paulo (FO-USP), São Paulo, Brazil: Blackwell Munksgaard; 2018;53:288–97. |
| 167. Muniz FWMG, Taminski K, Cavagni J, Celeste RK, Weidlich P, Rosing CK. The effect of statins on periodontal treatment-a systematic review with meta-analyses and meta-regression. Clin Oral Investig. Germany; 2018;22:671–87. |
| 168. Muthukuru M, Zainvi A, Esplugues EO, Flemmig TF. Non-surgical therapy for the management of peri-implantitis: a systematic review. Clin Oral Implants Res. Denmark; 2012;23 Suppl 6:77–83. |
| 169. Needleman I, Suvan J, Moles DR, Pimlott J. A systematic review of professional mechanical plaque removal for prevention of periodontal diseases. J Clin Periodontol. United States; 2005;32 Suppl 6:229–82. |
| 170. Ng E, Byun R, Spahr A, Divnic-Resnik T. The efficacy of air polishing devices in supportive periodontal therapy: A systematic review and meta-analysis. Quintessence Int. Germany; 2018;49:453–67. |
| 171. Nibali L, Koidou VP, Hamborg T, Donos N. Empirical or microbiologically guided systemic antimicrobials as adjuncts to non‐surgical periodontal therapy? A systematic review. J Clin Periodontol. 2019;46:999–1012. |
| 172. Nicolini ACAC, Grisa TA, Muniz FWMGFWMG, Rosing CK, Cavagni J, Rösing CK, et al. Effect of adjuvant use of metformin on periodontal treatment: a systematic review and meta-analysis. Clin Oral Investig. Germany; 2019;23:2659–66. |

Additional file 3. continued

| 173. O’Rourke VJ. Azithromycin as an adjunct to non-surgical periodontal therapy: a systematic review. Aust Dent J. Australia; 2017;62:14–22. |
| --- |
| 174. Oates TW, Robinson M, Gunsolley JC. Surgical therapies for the treatment of gingival recession. A systematic review. Ann Periodontol. United States; 2003;8:303–20. |
| 175. Paraskevas S, van der Weijden GA. A review of the effects of stannous fluoride on gingivitis. J Clin Periodontol. United States; 2006;33:1–13. |
| 176. Parrish LC, Miyamoto T, Fong N, Mattson JS, Cerutis DR. Non-bioabsorbable vs. bioabsorbable membrane: assessment of their clinical efficacy in guided tissue regeneration technique. A systematic review. J Oral Sci. Japan; 2009;51:383–400. |
| 177. Pavia M, Nobile CGA, Angelillo IF. Meta-analysis of local tetracycline in treating chronic periodontitis. J Periodontol. Medical School, Univ. Catanzaro Magna Græcia, Via Tommaso Campanella, 88100 Catanzaro, Italy: American Academy of Periodontology; 2003;74:916–32. |
| 178. Pavia M, Nobile CGA, Bianco A, Angelillo IF. Meta-analysis of local metronidazole in the treatment of chronic periodontitis. J Periodontol. Medical School, Univ. of Catanzaro Magna Graecia, Via Tommaso Campanella, 88100 Catanzaro, Italy; 2004;75:830–8. |
| 179. Plachokova AS, Nikolidakis D, Mulder J, Jansen JA, Creugers NHJ. Effect of platelet-rich plasma on bone regeneration in dentistry: a systematic review. Clin Oral Implants Res. Denmark; 2008;19:539–45. |
| 180. Polak D, Martin C, Sanz-Sanchez I, Beyth N, Shapira L. Are anti-inflammatory agents effective in treating gingivitis as solo or adjunct  therapies? A systematic review. J Clin Periodontol. United States; 2015;42 Suppl 1:S139-51. |
| 181. Preshaw PM, Hefti AF, Bradshaw MH. Adjunctive subantimicrobial dose doxycycline in smokers and non-smokers with chronic periodontitis. J Clin Periodontol. School of Dental Sciences, Newcastle University, United Kingdom; 2005;32:610–6. |
| 182. Qadri T, Javed F, Johannsen G, Gustafsson A. Role of diode lasers (800-980 nm) as adjuncts to scaling and root planing in the  treatment of chronic periodontitis: a systematic review. Photomed Laser Surg. United States; 2015;33:568–75. |
| 183. Rabelo CC, Feres M, Gonçalves C, Figueiredo LC, Faveri M, Tu Y-K, et al. Systemic antibiotics in the treatment of aggressive periodontitis. A systematic review and a Bayesian Network meta-analysis. J Clin Periodontol. 2015;42:647–57. |
| 184. Rajendra A, Spivakovsky S. Antibiotics in aggressive periodontitis, is there a clinical benefit? Evid Based Dent. England; 2016;17:100–100. |
| 185. Ravida A, Wang I-CI-C, Barootchi S, Askar H, Tavelli L, Gargallo-Albiol J, et al. Meta-analysis of randomized clinical trials comparing clinical and patient-reported outcomes between extra-short (≤6 mm) and longer (≥10 mm) implants. J Clin Periodontol. United States; 2019;46:118–42. |
| 186. Renatus A. Clinical Efficacy of Azithromycin as an Adjunctive Therapy to Non-Surgical Periodontal Treatment of Periodontitis: A Systematic Review and Meta-Analysis. J Clin DIAGNOSTIC Res. 2016;10:ZE01–7. |
| 187. Ricci L, Perrotti V, Ravera L, Scarano A, Piattelli A, Iezzi G. Rehabilitation of deficient alveolar ridges using titanium grids before and simultaneously with implant placement: a systematic review. J Periodontol. United States; 2013;84:1234–42. |
| 188. Rocchietta I, Fontana F, Simion M. Clinical outcomes of vertical bone augmentation to enable dental implant placement: a systematic review. J Clin Periodontol. United States; 2008;35:203–15. |
| 189. Roccuzzo M, Bunino M, Needleman I, Sanz M. Periodontal plastic surgery for treatment of localized gingival recessions: a systematic review. J Clin Periodontol. United States; 2002;29 Suppl 3:176–8. |

Additional file 3. continued

| 190. Rojas MA, Marini L, Pilloni A, Sahrmann P. Early wound healing outcomes after regenerative periodontal surgery with enamel matrix derivatives or guided tissue regeneration: a systematic review. BMC Oral Health. England; 2019;19:76. |
| --- |
| 191. Roncati M, Gariffo A. Systematic review of the adjunctive use of diode and Nd:YAG lasers for nonsurgical periodontal instrumentation. Photomed Laser Surg. United States; 2014;32:186–97. |
| 192. Rovai ES, Souto MLS, Ganhito JA, Holzhausen M, Chambrone L, Pannuti CM. Efficacy of Local Antimicrobials in the Non-Surgical Treatment of Patients With Periodontitis and Diabetes: A Systematic Review. J Periodontol. United States; 2016;87:1406–17. |
| 193. Sahrmann P, Puhan MA, Attin T, Schmidlin PR. Systematic review on the effect of rinsing with povidone-iodine during nonsurgical periodontal therapy. J Periodontal Res. United States; 2010;45:153–64. |
| 194. Sahrmann P, Attin T, Schmidlin PR. Regenerative treatment of peri-implantitis using bone substitutes and membrane: a systematic review. Clin Implant Dent Relat Res. United States; 2011;13:46–57. |
| 195. Salvi GE, Ramseier CA. Efficacy of patient-administered mechanical and/or chemical plaque control protocols in the management of peri-implant mucositis. A systematic review. J Clin Periodontol. United States; 2015;42 Suppl 1:S187-201. |
| 196. Sälzer S, Slot DE, Dörfer C, Van der Weijden GA, Salzer S, Slot DE, et al. Comparison of triclosan and stannous fluoride dentifrices on parameters of gingival inflammation and plaque scores: A systematic review and meta-analysis. Int J Dent Hyg. Clinic for Conservative Dentistry and Periodontology, School for Dental Medicine, Christian-Albrechts-University Kiel, Kiel, Germany, England: Blackwell Publishing Inc.; 2015;13:1–17. |
| 197. Sanz-Sánchez I, Oteo-Calatayud J, Serrano J, Martín C, Herrera D. Changes in plaque and gingivitis levels after tooth bleaching: A systematic review. Int J Dent Hyg. ETEP (Etiology and Therapy of Periodontal Diseases) Research Group, University Complutense, Madrid, Spain: Blackwell Publishing Inc.; 2019;17:117–29. |
| 198. Sanz-Sánchez I, Sanz-Martín I, de Albornoz A, Figuero E, Sanz M. Biological effect of the abutment material on the stability of peri-implant marginal bone levels: A systematic review and meta-analysis. Clin Oral Implants Res. 2018;29:124–44. |
| 199. Schmidt JC, Walter C, Rischewski JR, Weiger R. Treatment of periodontitis as a manifestation of neutropenia with or without systemic antibiotics: a systematic review. Pediatr Dent. United States; 2013;35:E54-63. |
| 200. Schwarz F, Aoki A, Becker J, Sculean A. Laser application in non-surgical periodontal therapy: A systematic review. J Clin Periodontol. 2008;35:29–44. |
| 201. Schwarz F, Becker K, Sager M. Efficacy of professionally administered plaque removal with or without adjunctive measures for the treatment of peri-implant mucositis. A systematic review and meta-analysis. J Clin Periodontol. 2015;42:S202–13. |
| 202. Sculean A, Nikolidakis D, Schwarz F. Regeneration of periodontal tissues: combinations of barrier membranes and grafting materials - biological foundation and preclinical evidence: a systematic review. J Clin Periodontol. United States; 2008;35:106–16. |
| 203. Serrano J, Escribano M, Roldan S, Martin C, Herrera D. Efficacy of adjunctive anti-plaque chemical agents in managing gingivitis: a systematic review and meta-analysis. J Clin Periodontol. United States; 2015;42 Suppl 1:S106-38. |

Additional file 3. continued

| 204. Sgolastra F, Severino M, Pietropaoli D, Gatto R, Monaco A, Petrucci A, et al. Effectiveness of periodontal treatment to improve metabolic control in patients with chronic periodontitis and type 2 diabetes: A meta-analysis of randomized clinical trials. J Periodontol. Department of Health Sciences, School of Dentistry, University of L’Aquila, Via Vetoio 1, 67100 L’Aquila, Italy, United States; 2013;84:958–73. |
| --- |
| 205. Sgolastra F, Petrucci A, Gatto R, Giannoni M, Monaco A. Long-term efficacy of subantimicrobial-dose doxycycline as an adjunctive treatment to scaling and root planing: a systematic review and meta-analysis. J Periodontol. United States; 2011;82:1570–81. |
| 206. Sgolastra F, Petrucci A, Gatto R, Monaco A. Efficacy of Er:YAG laser in the treatment of chronic periodontitis: systematic review and meta-analysis. Lasers Med Sci. England; 2012;27:661–73. |
| 207. Sgolastra F, Petrucci A, Gatto R, Monaco A, Petrucci A, Monaco A. Effectiveness of systemic amoxicillin/metronidazole as an adjunctive therapy to full-mouth scaling and root planing in the treatment of aggressive periodontitis: a systematic review and meta-analysis. J Periodontol. United States; 2012;83:1257–69. |
| 208. Sgolastra F, Petrucci A, Severino M, Graziani F, Gatto R, Monaco A. Adjunctive photodynamic therapy to non-surgical treatment of chronic periodontitis: a systematic review and meta-analysis. J Clin Periodontol. United States; 2013;40:514–26. |
| 209. Sgolastra F, Severino M, Gatto R, Monaco A. Effectiveness of diode laser as adjunctive therapy to scaling root planning in the treatment of chronic periodontitis: a meta-analysis. Lasers Med Sci. England; 2013;28:1393–402. |
| 210. Sgolastra F, Severino M, Petrucci A, Gatto R, Monaco A. Nd:YAG laser as an adjunctive treatment to nonsurgical periodontal therapy: a meta-analysis. Lasers Med Sci. England; 2014;29:887–95. |
| 211. Sicilia A, Arregui I, Gallego M, Cabezas B, Cuesta S. A systematic review of powered vs manual toothbrushes in periodontal cause-related therapy. J Clin Periodontol. United States; 2002;29 Suppl 3:31–9. |
| 212. Silveira GS, de Almeida NV, Pereira DMT, Mattos CT, Mucha JN. Prosthetic replacement vs space closure for maxillary lateral incisor agenesis: A systematic review. Am J Orthod Dentofacial Orthop. United States; 2016;150:228–37. |
| 213. Sinjab K, Zimmo N, Lin G-H, Chung M-P, Shaikh L, Wang H-L. The Effect of Locally Delivered Statins on Treating Periodontal Intrabony Defects: A Systematic Review and Meta-Analysis. J Periodontol. United States; 2017;88:357–67. |
| 214. Sivaramakrishnan G, Sridharan K. Photodynamic therapy for the treatment of peri-implant diseases: A network meta-analysis of randomized controlled trials. Photodiagnosis Photodyn Ther. Netherlands; 2018;21:1–9. |
| 215. Slot DE, Berchier CE, Addy M, Van der Velden U, Van der Weijden GA. The efficacy of chlorhexidine dentifrice or gel on plaque, clinical parameters of gingival inflammation and tooth discoloration: a systematic review. Int J Dent Hyg. England; 2014;12:25–35. |
| 216. Slot DE, Dorfer CE, Van der Weijden GA. The efficacy of interdental brushes on plaque and parameters of periodontal inflammation: a systematic review. Int J Dent Hyg. England; 2008;6:253–64. |
| 217. Slot DE, Koster TJG, Paraskevas S, Van der Weijden GA. The effect of the Vector scaler system on human teeth: a systematic review. Int J Dent Hyg. England; 2008;6:154–65. |
| 218. Slot DE, De Geest S, Van Der Weijden FA, Quirynen M. Treatment of oral malodour. Medium-term efficacy of mechanical and/or chemical agents: A systematic review. J Clin Periodontol. 2015;42:S303–16. |

Additional file 3. continued

| 219. Slot DE, Jorritsma KH, Cobb CM, Van der Weijden FA. The effect of the thermal diode laser (wavelength 808-980 nm) in non-surgical periodontal therapy: a systematic review and meta-analysis. J Clin Periodontol. United States; 2014;41:681–92. |
| --- |
| 220. Slot DE, Kranendonk AA, Paraskevas S, Van der Weijden F. The effect of a pulsed Nd:YAG laser in non-surgical periodontal therapy. J Periodontol. United States; 2009;80:1041–56. |
| 221. Smiley CJ, Tracy SL, Abt E, Michalowicz BS, John MT, Gunsolley J, et al. Systematic review and meta-analysis on the nonsurgical treatment of chronic periodontitis by means of scaling and root planing with or without adjuncts. J Am Dent Assoc. 2015;146:508-524.e5. |
| 222. Souto MLS, Rovai ES, Ganhito JA, Holzhausen M, Chambrone L, Pannuti CM. Efficacy of systemic antibiotics in nonsurgical periodontal therapy for diabetic subjects: a systematic review and meta-analysis. Int Dent J. 2018;68:207–20. |
| 223. Stoecklin-Wasmer C, Rutjes AWS, da Costa BR, Salvi GE, Juni P, Sculean A. Absorbable collagen membranes for periodontal regeneration: a systematic review. J Dent Res. United States; 2013;92:773–81. |
| 224. Stoeken JE, Paraskevas S, van der Weijden GA. The Long-Term Effect of a Mouthrinse Containing Essential Oils on Dental Plaque and Gingivitis: A Systematic Review. J Periodontol. United States; 2007;78:1218–28. |
| 225. Supranoto S, Slot D, Addy M, Van der Weijden G. The effect of chlorhexidine dentifrice or gel versus chlorhexidine mouthwash on plaque, gingivitis, bleeding and tooth discoloration: a systematic review. Int J Dent Hyg. 2015;13:83–92. |
| 226. Tabassum S, Adnan S, Khan FR. Gingival Retraction Methods: A Systematic Review. J Prosthodont. United States; 2017;26:637–43. |
| 227. Tan WC, Lang NP, Zwahlen M, Pjetursson BE. A systematic review of the success of sinus floor elevation and survival of implants inserted in combination with sinus floor elevation Part II: Transalveolar technique. J Clin Periodontol. 2008;35:241–54. |
| 228. Tassi SA, Sergio NZ, Misawa MYO, Villar CC. Efficacy of stem cells on periodontal regeneration: Systematic review of pre-clinical studies. J Periodontal Res. United States; 2017;52:793–812. |
| 229. Tatakis DN, Chambrone L. The Effect of Suturing Protocols on Coronally Advanced Flap Root-Coverage Outcomes: A Meta-Analysis. J Periodontol. 2016;87:148–55. |
| 230. Teeuw WJ, Slot DE, Susanto H, Gerdes VEA, Abbas F, D’Aiuto F, et al. Treatment of periodontitis improves the atherosclerotic profile: A systematic review and meta-analysis. J Clin Periodontol. 2014;41:70–9. |
| 231. Teeuw WJ, Gerdes VEA, Loos BG. Effect of periodontal treatment on glycemic control of diabetic patients: a systematic review and meta-analysis. Diabetes Care. United States; 2010;33:421–7. |
| 232. Thoma DS, Bienz SP, Figuero E, Jung RE, Sanz-Martín I. Efficacy of lateral bone augmentation performed simultaneously with dental implant placement: A systematic review and meta-analysis. J Clin Periodontol. 2019;46:257–76. |
| 233. Troiano G, Lo Russo L, Canullo L, Ciavarella D, Lo Muzio L, Laino L. Early and late implant failure of submerged versus non-submerged implant healing: A systematic review, meta-analysis and trial sequential analysis. J Clin Periodontol. 2018;45:613–23. |
| 234. Trombelli L, Farina R. Clinical outcomes with bioactive agents alone or in combination with grafting or guided tissue regeneration. J Clin Periodontol. United States; 2008;35:117–35. |
| 235. Trombelli L, Heitz-Mayfield LJA, Needleman I, Moles D, Scabbia A. A systematic review of graft materials and biological agents for periodontal intraosseous defects. J Clin Periodontol. United States; 2002;29 Suppl 3:112–7. |

Additional file 3. continued

| 236. Tunkel J, Heinecke A, Flemmig TF. A systematic review of efficacy of machine-driven and manual subgingival debridement in the treatment of chronic periodontitis. J Clin Periodontol. United States; 2002;29 Suppl 3:71–2. |
| --- |
| 237. Uppal A, Uppal S, Pinto A, Dutta M, Shrivatsa S, Dandolu V, et al. The effectiveness of periodontal disease treatment during pregnancy in reducing the risk of experiencing preterm birth and low birth weight A meta-analysis. J Am Dent Assoc. Oral Medicine, School of Dental Medicine, University of Pennsylvania, Philadelphia, United States: American Dental Association; 2010;141:1423–34. |
| 238. Valkenburg C, Slot DE, Bakker EWP, Van der Weijden FA. Does dentifrice use help to remove plaque? A systematic review. J Clin Periodontol. United States; 2016;43:1050–8. |
| 239. Van der Sleen MI, Slot DE, Van Trijffel E, Winkel EG, Van der Weijden GA, der Sleen MI, et al. Effectiveness of mechanical tongue cleaning on breath odour and tongue coating: a systematic review. Int J Dent Hyg. England; 2010;8:258–68. |
| 240. Van der Sluijs M, Van der Sluijs E, Van der Weijden F, Slot DE, der Sluijs M, der Sluijs E, et al. The effect on clinical parameters of periodontal inflammation following non-surgical periodontal therapy with ultrasonics and chemotherapeutic cooling solutions: a systematic review. J Clin Periodontol. United States; 2016;43:1074–85. |
| 241. Van der Weijden FA, Campbell SL, Dorfer CE, Gonzalez-Cabezas C, Slot DE. Safety of oscillating-rotating powered brushes compared to manual toothbrushes: a systematic review. J Periodontol. United States; 2011;82:5–24. |
| 242. van der Weijden GA, Hioe KPK. A systematic review of the effectiveness of self-performed mechanical plaque removal in adults with gingivitis using a manual toothbrush. J Clin Periodontol. United States; 2005;32 Suppl 6:214–28. |
| 243. Van Leeuwen MPC, Slot DE, Van der Weijden GA. The effect of an essential-oils mouthrinse as compared to a vehicle solution on plaque and gingival inflammation: a systematic review and meta-analysis. Int J Dent Hyg. England; 2014;12:160–7. |
| 244. Van Leeuwen MPC, Slot DE, Van Der Weijden GA. Essential oils compared to chlorhexidine with respect to plaque and parameters of gingival inflammation: A systematic review. J Periodontol. 2011;82:174–94. |
| 245. Van Strydonck DAC, Slot DE, Van der Velden U, Van der Weijden F. Effect of a chlorhexidine mouthrinse on plaque, gingival inflammation and staining in gingivitis patients: a systematic review. J Clin Periodontol. United States; 2012;39:1042–55. |
| 246. Vives-Soler A, Chimenos-Kustner E. Effect of probiotics as a complement to non-surgical periodontal therapy in chronic periodontitis: a systematic review. Med Oral Patol Oral Cir Bucal. Spain; 2020;25:e161–7. |
| 247. Vohra F, Al-Kheraif AA, Almas K, Javed F. Comparison of crestal bone loss around dental implants placed in healed sites using flapped and flapless techniques: A systematic review. J Periodontol. 2015;86:185–91. |
| 248. Vohra F, Akram Z, Safii SH, Vaithilingam RD, Ghanem A, Sergis K, et al. Role of antimicrobial photodynamic therapy in the treatment of aggressive periodontitis: A systematic review. Photodiagnosis Photodyn Ther. 2016;13:139–47. |
| 249. Wambier LM, de Geus JL, Boing TF, Chibinski ACR, Wambier DS, Rego RO, et al. Intrapocket topical anesthetic versus injected anesthetic for pain control during scaling and root planing in adult patients: Systematic review and meta-analysis. J Am Dent Assoc. England; 2017;148:814-824.e2. |

Additional file 3. continued

| 250. Wambier LM, de Geus JL, Chibinski ACR, Wambier DS, Rego RO, Loguercio AD, et al. Intra-pocket anaesthesia and pain during probing, scaling and root planing: a systematic review and meta-analysis. J Clin Periodontol. 2016;43:754–66. |
| --- |
| 251. Wang T-F, Jen I-A, Chou C, Lei Y-P. Effects of periodontal therapy on metabolic control in patients with type 2 diabetes mellitus and periodontal disease: a meta-analysis. Medicine (Baltimore). United States; 2014;93:e292. |
| 252. Wang X, Han X, Guo X, Luo X, Wang D. The effect of periodontal treatment on hemoglobin a1c levels of diabetic patients: a systematic review and meta-analysis. PLoS One. United States; 2014;9:e108412. |
| 253. Wang Y, Fan F, Li X, Zhou Q, He B, Huang X, et al. Influence of gingival retraction paste versus cord on periodontal health: a systematic review and meta-analysis. Quintessence Int. Germany; 2019;50:234–44. |
| 254. West NX, Seong J, Davies M. Management of dentine hypersensitivity: efficacy of professionally and self-administered agents. J Clin Periodontol. United States; 2015;42 Suppl 1:S256-302. |
| 255. Wu Y-C, Lin L-K, Song C-J, Su Y-X, Tu Y-K. Comparisons of periodontal regenerative therapies: A meta-analysis on the long-term efficacy. J Clin Periodontol. United States; 2017;44:511–9. |
| 256. Xue D, Tang L, Bai Y, Ding Q, Wang P, Zhao Y. Clinical efficacy of photodynamic therapy adjunctive to scaling and root planing  in the treatment of chronic periodontitis: A systematic review and meta-analysis. Photodiagnosis Photodyn Ther. Netherlands; 2017;18:119–27. |
| 257. Xue D, Zhao Y. Clinical effectiveness of adjunctive antimicrobial photodynamic therapy for residual pockets during supportive periodontal therapy: A systematic review and meta-analysis. Photodiagnosis Photodyn Ther. Netherlands; 2017;17:127–33. |
| 258. Yap KCH, Pulikkotil SJ. Systemic doxycycline as an adjunct to scaling and root planing in diabetic patients with periodontitis: a systematic review and meta-analysis. BMC Oral Health. England; 2019;19:209. |
| 259. Ye P, Wei A, Wang Y, Cai Y-J. Autologous platelet concentrates as clinical substitutes for connective tissue graft in the treatment of Miller class I and II gingival recessions: An updated meta-analysis. Int J Periodontics Restor Dent. 2020;40:E53–63. |
| 260. Zandbergen D, Slot DE, Cobb CM, Van der Weijden FA. The clinical effect of scaling and root planing and the concomitant administration of systemic amoxicillin and metronidazole: a systematic review. J Periodontol. United States; 2013;84:332–51. |
| 261. Zandbergen D, Slot DE, Niederman R, Van der Weijden FA. The concomitant administration of systemic amoxicillin and metronidazole compared to scaling and root planing alone in treating periodontitis: =a systematic review=. BMC Oral Health. England; 2016;16:27. |
| 262. Zhang J, Ab Malik N, McGrath C, Lam O. The effect of antiseptic oral sprays on dental plaque and gingival inflammation:  A systematic review and meta-analysis. Int J Dent Hyg. England; 2019;17:16–26. |
| 263. Zhang Z, Zheng Y, Bian X. Clinical effect of azithromycin as an adjunct to non-surgical treatment of chronic periodontitis: A meta-analysis of randomized controlled clinical trials. J Periodontal Res. Department of Periodontology, Tianjin Stomatological Hospital, Nankai University, Tianjin, China: Blackwell Munksgaard; 2016;51:275–83. |
| 264. Zhao Y, Yin Y, Tao L, Nie P, Tang Y, Zhu M. Er:YAG laser versus scaling and root planing as alternative or adjuvant for chronic periodontitis treatment: a systematic review. J Clin Periodontol. 2014;41:1069–79. |
| 265. Zhou S, Sun C, Huang S, Wu X, Zhao Y, Pan C, et al. Efficacy of Adjunctive Bioactive Materials in the Treatment of Periodontal Intrabony Defects: A Systematic Review and Meta-Analysis. Biomed Res Int. United States; 2018;2018:8670832. |
