# Supplementary material for: There is still room for improvement in the completeness of abstract reporting according to the PRISMA-A checklist: a cross-sectional study on systematic reviews in periodontology
Source: BMC Med Res Methodol. 2021 Feb 11;21:33. doi: 10.1186/s12874-021-01223-y (PMC7879697; doi:10.1186/s12874-021-01223-y)
Supplement: Supplementary file 5 — Additional file 5. PRISMA-A item compliance by geographical area. [file 12874_2021_1223_MOESM5_ESM.docx]

**Additional file 5**: PRISMA-A items compliance by geographical area.

|  | **Asia (n=40)** | | **Europe (n=125)** | | **Latin America (n=38)** | | **North America (n=41)** | |  |
| --- | --- | --- | --- | --- | --- | --- | --- | --- | --- |
|  | **Mean** | **95% CI** | **Mean** | **95% CI** | **Mean** | **95% CI** | **Mean** | **95% CI** | **p-value** |
| **TITLE** | 97.50 | 92.44-100.0 | 93.25 | 89.02-97.47 | 93.42 | 86.61-100.0 | 87.80 | 77.34-98.26 | 0.409 |
| **OBJECTIVES** | 78.75 | 69.24-88.25 | 86.90 | 82.56-91.24 | 90.79 | 84.33-97.24 | 76.83 | 66.18-87.47 | 0.074 |
| **ELIGIBILITY CRITERIA** | 55.00 | 40.60-69.39 | 64.29 | 57.15-71.41 | 75.00 | 63.06-86.93 | 70.73 | 57.53-83.92 | 0.155 |
| **INFORMATION SOURCES** | 75.00 | 63.54-86.45 | 53.57 | 45.91-61.23 | 78.95 | 67.08-90.80 | 59.76 | 47.93-71.57 | **0.001**** |
| **RISK OF BIAS** | 13.75 | 2.89-24.60 | 14.29 | 8.83-19.73 | 23.68 | 10.04-37.32 | 17.07 | 5.57-28.56 | 0.623 |
| **INCLUDED STUDIES** | 73.75 | 64.17-83.32 | 64.68 | 58.67-70.69 | 77.63 | 68.51-86.75 | 73.75 | 64.17-83.32 | 0.122 |
| **SYNTHESIS OF RESULTS** | 87.50 | 80.48-94.51 | 72.22 | 65.95-78.48 | 75.00 | 63.69-86.30 | 87.80 | 79.32-96.28 | **0.013*** |
| **DESCRIPTION OF THE EFFECT** | 82.50 | 72.54-92.45 | 76.59 | 70.71-82.45 | 75.00 | 63.06-86.93 | 84.15 | 73.89-94.39 | 0.323 |
| **STRENGTHS AND LIMITATIONS OF THE EVIDENCE** | 17.50 | 6.91-28.08 | 36.11 | 28.57-43.64 | 31.58 | 18.11-45.04 | 39.02 | 24.68-53.36 | 0.076 |
| **INTERPRETATION** | 96.25 | 91.98-100.0 | 91.27 | 87.39-95.14 | 94.74 | 89.62-99.84 | 95.12 | 88.23-100.0 | 0.283 |
| **FUNDING AND CONFLICT OF INTEREST** | 0.00 | -- | 0.00 | -- | 0.00 | -- | 0.00 | -- | 1.000 |
| **REGISTRATION** | 0.00 | -- | 0.79 | 0.00-2.36 | 0.00 | -- | 0.00 | -- | 0.815 |
| **TOTAL PRISMA-A SCORE** | 56.46 | 53.42-59.49 | 54.50 | 52.48-56.52 | 59.52 | 56.00-63.03 | 57.52 | 55.18-59.85 | **0.035*** |

**Kruskal-Wallis´s** test *p<0.05; **p<0.01; ***p<0.001; *Categories involving less than 15 items have been excluded from the analysis*
